# The future of life expectancy and life expectancy inequalities in England and Wales: Bayesian spatiotemporal forecasting

**DOI:** 10.1016/S0140-6736(15)60296-3

**Published:** 2015-07-11

**Authors:** James E Bennett, Guangquan Li, Kyle Foreman, Nicky Best, Vasilis Kontis, Clare Pearson, Peter Hambly, Majid Ezzati

**Affiliations:** a Department of Epidemiology and Biostatistics, School of Public Health, and MRC-PHE Centre for Environment and Health, Imperial College London, London, UK; b UK Small Area Health Statistics Unit, Imperial College London, London, UK; c Department of Mathematics and Information Sciences, Northumbria University, Newcastle-upon-Tyne, UK; d GlaxoSmithKline, London, UK

## Abstract

**Background:**

To plan for pensions and health and social services, future mortality and life expectancy need to be forecast. Consistent forecasts for all subnational units within a country are very rare. Our aim was to forecast mortality and life expectancy for England and Wales' districts.

**Methods:**

We developed Bayesian spatiotemporal models for forecasting of age-specific mortality and life expectancy at a local, small-area level. The models included components that accounted for mortality in relation to age, birth cohort, time, and space. We used geocoded mortality and population data between 1981 and 2012 from the Office for National Statistics together with the model with the smallest error to forecast age-specific death rates and life expectancy to 2030 for 375 of England and Wales' 376 districts. We measured model performance by withholding recent data and comparing forecasts with this withheld data.

**Findings:**

Life expectancy at birth in England and Wales was 79·5 years (95% credible interval 79·5–79·6) for men and 83·3 years (83·3–83·4) for women in 2012. District life expectancies ranged between 75·2 years (74·9–75·6) and 83·4 years (82·1–84·8) for men and between 80·2 years (79·8–80·5) and 87·3 years (86·0–88·8) for women. Between 1981 and 2012, life expectancy increased by 8·2 years for men and 6·0 years for women, closing the female–male gap from 6·0 to 3·8 years. National life expectancy in 2030 is expected to reach 85·7 (84·2–87·4) years for men and 87·6 (86·7–88·9) years for women, further reducing the female advantage to 1·9 years. Life expectancy will reach or surpass 81·4 years for men and reach or surpass 84·5 years for women in every district by 2030. Longevity inequality across districts, measured as the difference between the 1st and 99th percentiles of district life expectancies, has risen since 1981, and is forecast to rise steadily to 8·3 years (6·8–9·7) for men and 8·3 years (7·1–9·4) for women by 2030.

**Interpretation:**

Present forecasts underestimate the expected rise in life expectancy, especially for men, and hence the need to provide improved health and social services and pensions for elderly people in England and Wales. Health and social policies are needed to curb widening life expectancy inequalities, help deprived districts catch up in longevity gains, and avoid a so-called grand divergence in health and longevity.

**Funding:**

UK Medical Research Council and Public Health England.

## Introduction

To plan for health and social services and pensions, forecasts of future mortality and life expectancy are needed.^1^, ^2^ These forecasts have been done for one or more countries.^3^, ^4^, ^5^, ^6^, ^7^, ^8^, ^9^, ^10^, ^11^ Consistent forecasts for all subnational units within a country are very rare,^12^ even though mortality and life expectancy vary substantially within countries, both geographically and in relation to social class. Planning and priority setting at the subnational level need local mortality forecasts. Local information is especially important in countries like the UK that are devolving health and social care responsibilities to local governments to ensure that such decentralisation, which might particularly reduce services in deprived areas, does not worsen health inequalities.

In this study, we used the methods of Bayesian (spatiotemporal) statistics to develop new approaches to forecasting of future mortality and life expectancy at a local, small-area level. We applied these methods to more than three decades of geocoded data from England and Wales' districts to estimate past trends and forecast future mortality and life expectancy by district.

## Methods

### Study design

We analysed trends from 1981 to 2012 and forecast age-specific death rates to 2030. The age groups in our analysis were 0 years, 1–4 years, 5 year age groups up to 84 years, and 85 years and older. We did not further divide the last age group because district population data were not further divided into age groups older than age 85 years before 1991. We used age-specific death rates to calculate life expectancy. Our analysis units were 375 of the 376 districts in England and Wales. We do not present estimates for the Isles of Scilly to comply with the Office for National Statistics (ONS) data disclosure policies for units with populations of less than 5000.

### Data

We used data for deaths in England and Wales between 1981 and 2012 (nearly 17 250 000 death records) held by the UK Small Area Health Statistics Unit and supplied by ONS. Data use was approved by the National Research Ethics Service (reference 12/LO/0566 and 12/LO/0567) and the National Information Governance Board and Ethics and Confidentiality Committee (approval for section 251 support [HRA—14/CAG/1039]). Age, sex, date of death, and postcode of residence were available for each record; we used postcodes to assign deaths to districts. Population data by age and sex for each district for 1981–2012 were from ONS. We also used ONS' district population projections with methods described elsewhere.^13^ Briefly, the present cohorts in each district are brought forward in time in 1 year increments and adjusted by district-level fertility, mortality, and migration; these demographic factors are themselves projected on the basis of recent trends.

### Statistical analysis

We used five forecasting models that were formulated to incorporate features of death rates in relation to age and birth cohort, and over time and space. Model specifications are provided in the appendix. We selected the model that had the smallest forecast error for reporting of results.

Death rates vary with age, and their age association tends to have a smooth pattern. Therefore, in models 1–4, we allowed each age group to have a different mortality level and trend, but modelled age group intercepts and slopes using a random walk structure that is widely used to characterise smoothly varying associations (appendix).^14^ This approach improves stability of death rates in each age group and avoids implausible age patterns of mortality that could occur if each age group is analysed separately.^7^, ^15^

Because time trends in death rates can be non-linear, we modelled time trends (of log-transformed death rates) using a linear term plus a smoothly varying non-linear term, specified with a random walk (model 1).^14^ Additionally, we formulated one of the models to have time trends that are faster or slower than linear through inclusion of an exponent on the trend term (model 2).

Different birth cohorts can have different mortality experiences—eg, risk factors like smoking or fetal or early-life determinants of health having cohort patterns. To take account of this characteristic, we formulated two of the models with a cohort component in trends, allowing trends to be more similar in adjacent birth cohorts than in those born in different eras using a random walk structure (models 3 and 4). One of these models (model 4) allowed the role of birth cohort to be more important in specific ages—eg, in older ages if mortality is affected by cumulative life-course risks, with use of an age-specific weight on the cohort term.

Finally, death rates, and change in death rates, might be more similar in neighbouring districts than in those farther away. We used the Besag, York, and Mollie spatial model, described in the appendix and elsewhere,^16^ which allows death rates and their trends in each district to be estimated on the basis of their own data and those of its neighbours. The extent to which neighbours share information depends on how uncertain death rates in each district are, and on the empirical similarity of neighbouring districts.

In addition to these four models, we implemented a model premised on the widely used Lee–Carter method, with the addition of a spatial component for district-level forecasting (model 5).^5^ We fitted models 1 and 3–5 with the Markov chain Monte Carlo algorithm in WinBUGS 1.4.3 and, to improve mixing, fitted model 2 in Stan 2.2.0, which uses an implementation of Hamiltonian Monte Carlo. We monitored convergence using trace plots and Brooks–Gelman–Rubin diagnostics,^17^ and collected 8000 postburn-in samples for inference and forecast. The reported 95% credible intervals represent the 2·5th to 97·5th percentiles of the posterior distribution of estimated death rates and life expectancies.

All analyses were sex-specific because mortality levels and trends differ by sex. We calculated national death rates for each age group as the population-weighted average of district death rates. We calculated life expectancies using life table methods.^18^ We used the Kannisto-Thatcher method to expand the terminal (85 years and older) age group of the life table.^19^ This method is designed for use in low-mortality populations and is used by the UN Population Division, WHO,^20^ the Human Mortality Database,^21^ and *The Lancet* Series on ageing.^22^ We calculated the contribution of specific age groups to life expectancy change using the so-called discrete method, as described elsewhere.^18^

**Table:** Life expectancy at birth by quintile of deprivation

	**Life expectancy in Q1 (least deprived; years)**	**Life expectancy in Q2 (years)**	**Life expectancy in Q3 (years)**	**Life expectancy in Q4 (years)**	**Life expectancy in Q5 (most deprived; years)**
**Men**					
1981	73·1 (73·0–73·1; 70·9–74·4)	72·5 (72·5–72·6; 69·4–74·0)	71·9 (71·8–71·9; 69·7–73·7)	71·1 (71·1–71·1; 69·5–73·4)	70·2 (70·2–70·2; 68·5–72·6)
2012	81·5 (81·5–81·6; 80·1–82·9)	80·7 (80·7–80·8; 79·4–82·2)	79·9 (79·8–79·9; 77·8–81·6)	79·0 (78·9–79·0; 77·5–83·4)	78·0 (78·0–78·1; 75·2–82·3)
2030	87·5 (85·9–89·3; 85·9–89·4)	86·7 (85·2–88·3; 85·6–88·4)	85·9 (84·3–87·7; 83·8–88·0)	85·1 (83·5–86·8; 83·7–90·7)	84·3 (82·7–86·1; 81·4–89·8)
**Women**					
1981	78·5 (78·4–78·5; 76·6–79·8)	78·1 (78·1–78·1; 74·1–79·8)	77·7 (77·6–77·7; 75·6–79·7)	77·3 (77·2–77·3; 75·6–80·5)	76·6 (76·5–76·6; 74·6–78·9)
2012	84·8 (84·7–84·9; 83·5–86·6)	84·2 (84·1–84·3; 82·6–86·1)	83·6 (83·6–83·7; 81·9–85·2)	82·9 (82·9–83·0; 81·2–87·3)	82·2 (82·1–82·3; 80·2–86·2)
2030	88·9 (87·9–90·2; 87·7–91·0)	88·3 (87·4–89·5; 86·7–90·6)	87·8 (86·8–88·9; 85·9–89·7)	87·1 (86·2–88·5; 85·5–92·6)	86·5 (85·5–87·7; 84·5–91·2)

Data are aggregate life expectancy, and those in parentheses are 95% credible interval; within-quintile range. Each district is assigned to a deprivation quintile on the basis of its 2011 Carstairs score, which combines information about unemployment, social class, crowding of housing, and (absence of) vehicle ownership. Q=quintile.

### Model performance

To assess performance of the forecasting models, we used the first 21 years of data (1981–2001) to estimate model parameters, which we then used to forecast for 2002–12, for which data were available but withheld. We compared forecasts from each model with the withheld data, and report forecast error (which measures systematic bias) and absolute forecast error (which measures any deviation from the data) for both life expectancy and age-specific death rates. Additionally, we report coverage of forecast uncertainty; if forecast death rates and their uncertainties are well estimated, estimated 90% credible intervals should cover 90% of the withheld data.

### Role of the funding source

The funder of the study had no role in study design, data collection, data analysis, data interpretation, or writing of the report. JEB, GL, VK, and PH had full access to all the data in the study and the corresponding author had final responsibility for the decision to submit for publication.

## Results

Detailed results on model performance are provided in the appendix. All five forecasting models had small in-sample errors—ie, their estimates were consistent with the observed data in years for which data were available and used in the model. However, forecast (out-of-sample) errors of the models differed, with the model that had a cohort component (model 3) having the smallest error. Forecasts from this model were almost unbiased (mean forecast error 0·01 years for both men and women). Their median absolute error was only 0·559 years (IQR 0·265–0·959) for men and 0·580 years (0·275–1·025) for women, better than that of the other four models. All other models underestimated future life expectancy, with mean forecast errors (ie, bias) ranging from 0·121 years for women in model 4 to 0·827 years for men in model 5 (appendix). We noted no systematic time trend to errors in model 3, whereas the bias and error in other models increased with increasing forecast period. Model 3 had the lowest mean forecast error in 12 (32%) of 38 age-sex groups and the lowest mean absolute forecast error in 16 (42%) of 38 age-sex groups, better than that of all other models. Results are therefore shown for model 3 hereafter. With very few exceptions, 90% coverage for all models and age groups ranged between 85% and 98%, with averages of between 92% and 94% for different models. Forecasts with coverages of greater than 90% might be viewed as slightly overcautious, allowing some more extreme outcomes to be considered than those that might actually occur.

Parameters for model 3, shown in the appendix, show that death rates in England and Wales had the well-known J-shaped age association, being high in the first year of life before falling rapidly and then rising gradually to old ages. For women, the rise in death rates was smooth throughout the whole age range; for men, a sharp increase occurred between 10–14 years and 15–19 years. This sex difference shows excess mortality in young men compared with women, which continues through middle ages.

**Figure 1:**
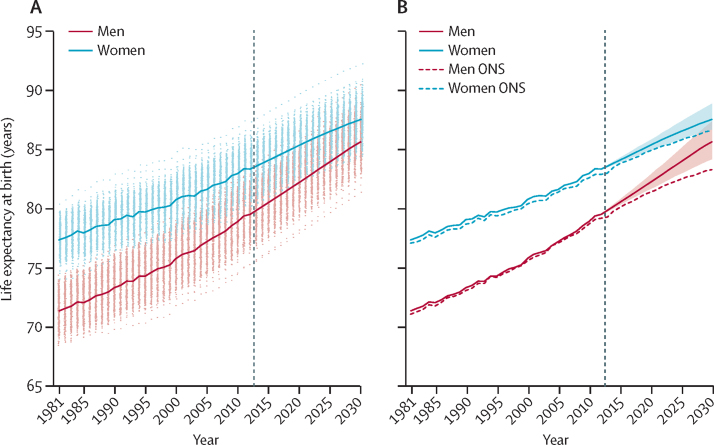
Trends and forecasts of (A) district and (B) national life expectancies The solid line in A shows national life expectancy and each point shows life expectancy for one district. In B, national life expectancy is shown with its 95% credible interval, together with life expectancy estimates and forecasts (principal variant) for England and Wales from ONS.^25^ The vertical dashed line shows when forecasts begin. The outlier district with high life expectancy is the City of London. The district of the City of London is geographically small and largely made up of offices for financial services companies. Its population was about 7500 in 2012 (compared with an average of around 149 000 in other districts), with very few residents being older than 65 years of age. Although the death rates are lower in the City of London than in other districts, its estimated death rates and life expectancy have much greater uncertainty than do those of other districts. ONS=Office for National Statistics.

**Figure 2:**
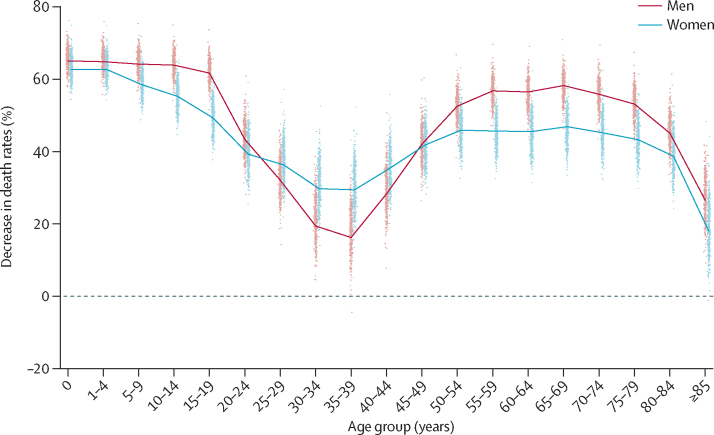
Decrease in death rates between 1981 and 2012 by age group in England and Wales' districts The solid line shows national decrease and each point shows change in one district. The dashed line represents no change in death rate.

Life expectancy at birth in England and Wales was 79·5 years (95% credible interval 79·5–79·6) for men and 83·3 years (83·3–83·4) for women in 2012 (figure 1). Between 1981 and 2012, life expectancy increased by 8·2 years for men and 6·0 years for women, closing the female–male gap from 6·0 years in 1981 to 3·8 years in 2012. The mortality reductions that led to this improvement were uneven across ages, with average reductions of 40% or more in middle and old ages, and even larger reductions in children and adolescents, but smaller reductions in young adults, especially in men (figure 2).

**Figure 3:**
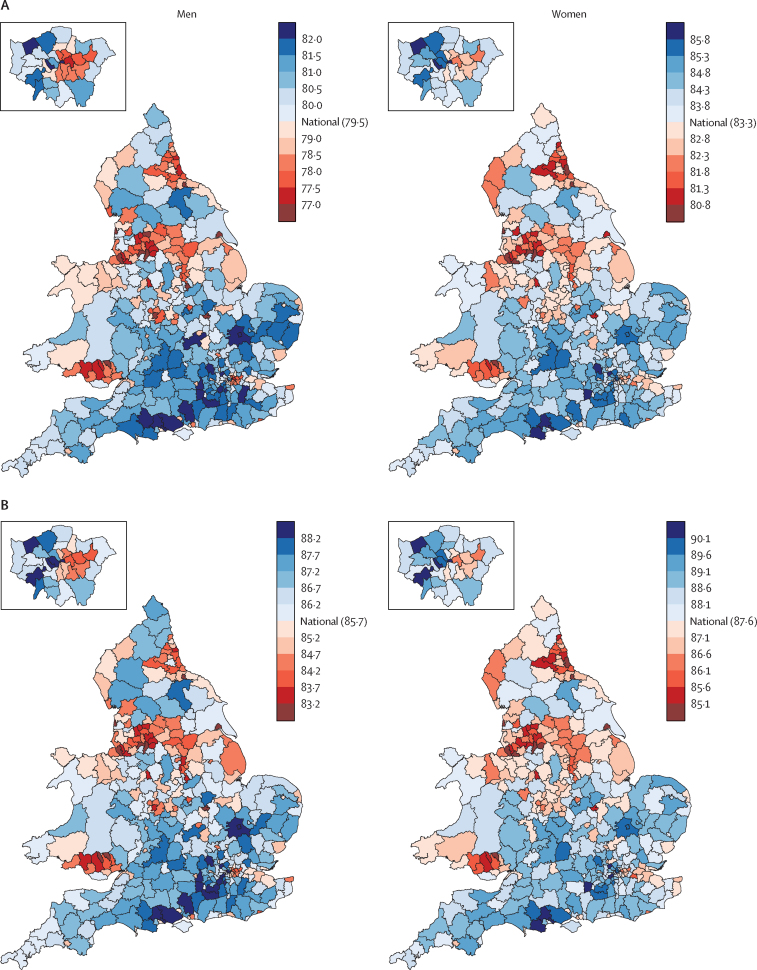
Life expectancy in England and Wales' districts in (A) 2012 and (B) 2030 The insets are enlarged views of London.

District life expectancy in 2012 ranged from 75·2 years (95% credible interval 74·9–75·6) to 83·4 years (82·1–84·8) for men, and from 80·2 years (79·8–80·5) to 87·3 years (86·0–88·8) for women (figure 3). The 8 year range for men is about the same as the difference between the UK and Sri Lanka or Vietnam in 2012; for women, the 7 year range is the same as the difference between the UK and Malaysia or Nicaragua.^23^ Life expectancy was lowest in urban northern England (including Blackpool, Liverpool, and Manchester) and southern Wales, and highest in southern England and some of London's more affluent districts. Within London, male and female life expectancies varied by 5–6 years between working-class Barking and Dagenham or Tower Hamlets (lowest) and the small district of City of London and wealthy Kensington and Chelsea (highest).

Between 1981 and 2012, district life expectancies increased by between 5·9 years and 11·3 years for men and between 4·0 years and 9·5 years for women. Life expectancy gains varied substantially across districts because large cross-district variations exist in how much age-specific death rates declined (figure 2). We noted no clear geographical pattern in life expectancy gain, with large and small gains seen in both the north and south of England and Wales (results not shown). Within London, male life expectancies in Kensington and Chelsea increased by 3·4 years (95% CI 2·7–4·1) more than in the deprived Lewisham, with female life expectancies increasing by 2·6 years (1·7–3·4) more. Life expectancies in 1981 and 2012 were moderately correlated (*r*=0·88 for men and 0·81 for women), but 30–40% of districts changed ranks by 50 or more places.

As a result of these trends, inequality in life expectancy across districts increased between 1981 and 2012. For example, the difference between the 1st and 99th percentiles of district life expectancies increased from 5·2 years (95% credible interval 5·0–5·3) to 6·1 years (5·9–6·4) for men, and from 4·5 years (4·3–4·7) to 5·6 years (5·3–6·0) for women between 1981 and 2012. Life expectancy was lower in more deprived districts, and the difference between the most and least deprived quintiles of districts increased from 2·8 years in 1981 to 3·5 years in 2012 for men, and from 1·9 years to 2·6 years for women (table). The inequality based on deprivation quintiles is smaller than total inequality due to within-quintile variations in life expectancy, which were the same size or larger than differences between the least and most deprived quintiles. Importantly, life expectancy varied substantially more across districts in the two most deprived quintiles than in the two least deprived ones (table).

**Figure 4:**
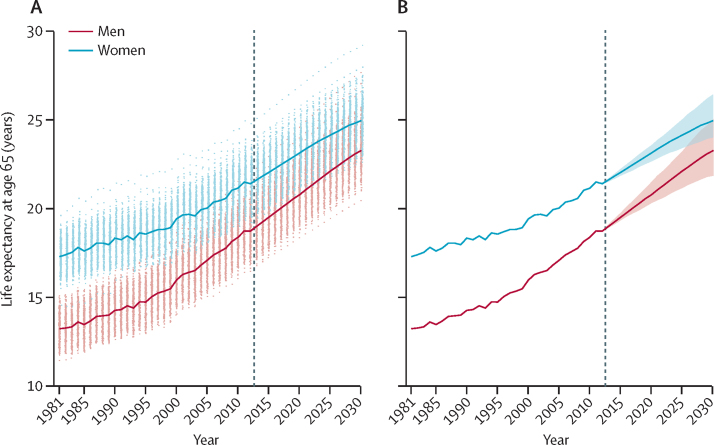
Trends and forecasts of (A) district and (B) national life expectancies at age 65 years The solid line in A shows national life expectancy and each point shows one district. In B, national life expectancy is shown with its 95% credible interval. The vertical dashed line shows when forecasts begin.

National life expectancy is expected to rise steadily and reach 85·7 years (95% credible interval 84·2–87·4) for men and 87·6 years (86·7–88·9) for women by 2030 (figure 1), shrinking the advantage of women compared with men to 1·9 years, less than a third of what it was in 1981. About two-thirds of the forecast gains in longevity in men and more than three-quarters of those in women will be due to better survival in those older than 65 years of age. Life expectancy at age 65 years will rise by 4·6 years (95% credible interval 3·1–6·3) for men and 3·5 years (2·5–4·9) for women (figure 4).

**Figure 5:**
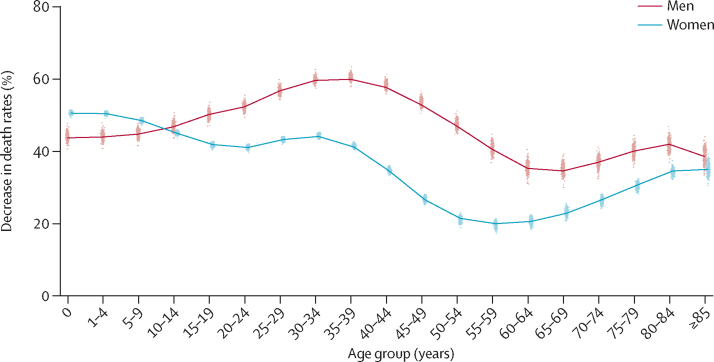
Forecast decrease in death rates between 2012 and 2030 by age group in England and Wales' districts The solid line shows decrease for England and Wales, and each point shows one district.

Life expectancy is forecast to increase in all districts, reaching or surpassing 81·4 years for men and reaching or surpassing 84·5 years for women in every district by 2030 (figure 1). In a few districts, life expectancy is expected to approach or pass 90 years for men and 92 years for women. The larger rise in male life expectancy than that of women will occur because death rates are forecast to fall more in men than in women, with a noticeable improvement in mortality of young and middle-aged adult men (figure 5). By contrast, death rates in middle-aged and old women are estimated to decrease more slowly in the future than they did during the past three decades. In a few districts, forecast male life expectancy will be almost the same as that of women in 2030, whereas in 2012, the female advantage was at least 2·4 years in every district.

Life expectancy inequalities are expected to continue to rise, with the difference between the 1st and 99th percentiles of district life expectancies reaching 8·3 years (95% credible interval 6·8–9·7) in men and 8·3 years (7·1–9·4) in women in 2030. The continued rise in inequality will be due to increasing variation within each deprivation quintile, whereas the differences between quintiles persist. Geographically, life expectancy will generally remain higher in the southern districts, with a slight weakening of the decreasing life expectancy gradient from south to north for men.

## Discussion

Our innovative subnational mortality forecasting predicts that life expectancy will continue to rise in England and Wales both nationally and in each district. Forecast national life expectancies in 2030 are 85·7 years for men and 87·6 years for women. For comparison, the highest national life expectancies worldwide in 2012 were 81·2 years for men in Iceland and 87·0 years for Japanese women.^23^ Therefore, female life expectancy in England and Wales in 2030 will be only slightly better than that of Japanese women in 2012. Most of the gains in longevity will be in those older than 65 years of age, and are, hence, highly relevant for planning pensions and health and social services. We also forecast that the closing of the female–male life expectancy gap will continue steadily. This narrowing will occur because death rates in middle-aged and old women are estimated to decrease more slowly in the future than they did in the past, perhaps partly due to accumulation of risks from smoking in middle-aged and older women.^24^

Life expectancy inequality is forecast to continue to rise across districts, however, with present and future inequalities partly related to district deprivation and partly associated with variation within deprivation quintiles, especially within the deprived quintiles. Furthermore, we found that life expectancy varied more in the more deprived quintiles, perhaps because deprived communities are more vulnerable to factors that affect health and longevity, but vary somewhat independently of deprivation.

Our national forecasts of life expectancy in 2030 are higher than those by ONS, by 2·4 years for men and 1·0 year for women.^25^ This difference might be because ONS extrapolates past trends in death rates, an approach that, as seen in the appendix, underestimates gains in life expectancy.^1^ Our subnational forecasts cannot be compared with those of previous studies because consistent small-area forecasting of population health is very rare (panel). Our subnational results have both similarities with and differences to the international scientific literature on life expectancy and longevity. The highest life expectancy in the world has risen steadily for decades, although the country that holds the top position has changed.^26^ Life expectancies of 90 years and older are therefore well within ranges that most demographers deem feasible. Some investigators have noted shrinking cross-country differences in life expectancy, and have advocated a worldwide so-called grand convergence in health,^27^ although others have noted a divergence, especially for adults.^28^, ^29^, ^30^ Our results show that national progress in the UK has come at the cost of rising within-country inequality, as also seen in the USA.^31^ If within-country divergences accompany aggregate gains elsewhere, poor health and health inequalities in the world will be associated more with community (and individual) characteristics than with national boundaries and characteristics. This possibility should motivate steps to make within-country inequalities part of worldwide health accounting systems.

Panel **Research in context**

**Systematic review**

We searched PubMed for articles published up to Feb 6, 2015, with no language restrictions, using the search terms “mortality” OR “longevity”, “forecasting”, and “spatial” OR “subnational” in the publication title and abstract. We also used a review of forecasting methods^7^ to identify mortality forecasting methods. Many attempts have been done to forecast mortality and life expectancy for national populations.^3^, ^4^, ^5^, ^6^, ^7^, ^8^, ^9^, ^10^, ^11^ Consistent forecasts for all subnational units within a country are, however, very rare, and tend to be for short periods.^12^ ONS projects mortality and life expectancy for England and Wales (but not its districts).^25^ Briefly, ONS “mortality projections are based largely on extrapolation of past trends in rates of mortality improvement. Expert opinion is used to inform the assumptions made about future mortality rates”.^25^ One of the assumptions that ONS used in the 2012-based projections is that the annual rates of decrease in death rates will converge to 1·2% for most ages in 2037, at which it will remain thereafter.

**Interpretation**

We used the methods of Bayesian (spatiotemporal) statistics to develop new models to forecast mortality and life expectancy at the district level in England and Wales. We formulated the models to incorporate important features of death rates in relation to age and birth cohort, and over time and space. Our national life expectancy forecasts are higher than those of ONS. Inequalities in life expectancy across districts have increased over time and are forecast to rise steadily. Our study provides an innovative methodological framework for subnational mortality forecasting and information for subnational health and social service policies.

The strengths of our study are its innovative scope of subnational forecasts with high spatial resolution; use of different forecasting models based on characteristics of death rates, and their patterns over age, birth cohort, time, and space, for coherent and unbiased forecasts; and rigorous testing of model performance. The key limitation of our work, shared by all other attempts to forecast the future, is the inability to account for unexpected events and major changes in social and health systems determinants of health, which can fundamentally change trends and, in extreme cases, even lead to a reversal of life expectancy gain. We forecast mortality and life expectancy at the district level because administrative units like districts are consistent over time and used for resource allocation and policy implementation. However, people who live in each district might change because of migration (within the country and overseas).^32^, ^33^, ^34^, ^35^ Therefore, life expectancy trends should not be attributed solely to changes in health status of individuals. Nonetheless, findings from studies in the UK^34^, ^35^, ^36^ and elsewhere^31^ have shown that migration is not sufficient to explain trends in health and health inequalities, and that these trends are largely due to changes in population health. Even if rising inequalities are partly due to migration, often by healthy people, from one area to another, such migration patterns have social and economic roots that should be addressed through employment opportunities, affordable housing, and high-quality education and health care.^37^, ^38^ We could not further divide the oldest (85 years or older) age group because geocoded population data were not available for age groups older than 85 years for some years. We accounted for this data limitation by using a life table method designed for low-mortality ageing populations. We measured deprivation at the district level, which was our unit of analysis. However, within-district variations exist in socioeconomic status. Finally, we did not forecast cause-specific mortality, which might be relevant for planning of health services, and should be the subject of future research.

Our higher forecast life expectancy than that of ONS means that pensions will have larger pay-outs than those currently planned, and health and social services will have to serve an even older population, with chronic and comorbid disorders, than that currently planned. National and subnational life expectancy gains will, however, come at the cost of rising inequalities, as has been the case for the past few decades. An implication of rising social inequalities in life expectancy is that better-off social groups, who are expected to live increasingly longer than will the more disadvantaged groups, will use health and social services for a longer time, creating a regressive transfer of resources.

Research in the UK has identified social policies in the 1970s and 1980s, which diminished job security, increased unemployment, and worsened economic inequalities, as important determinants of health inequalities.^39^ Our results show that life expectancy inequalities have increased steadily since this period, a trend that is expected to continue. Furthermore, the present UK coalition Government has cut public spending on a range of social determinants of health under the rhetoric of austerity.^40^ Such policies will, at best, cause the rising inequality trends to continue, and could well worsen them because their adverse effects are particularly large on children, working-age people, and disadvantaged social groups and communities, with signs of a rise in poverty already emerging.^40^

Access to high-quality health care can help reduce health inequalities through both preventive and lifesaving acute treatments. In the UK, use of general practice and many hospital services has been the same or even higher in people living in more deprived areas or from poorer socioeconomic groups, although inequalities might exist in use of some secondary care services and quality of care.^41^, ^42^, ^43^, ^44^, ^45^, ^46^, ^47^ Therefore universal health care through the National Health Service is likely to have had, and continue to have, an important role in limiting and reducing of health inequalities.^48^ However, parallel to worsening of social determinants of health, National Health Service reforms, which devolve health and social care responsibilities to local governments, coupled with tight budgets and an expanding role for the private sector in commissioning and provision of health services, will weaken health systems and worsen inequalities in health care access and quality.^49^, ^50^, ^51^ Rigorous comparative analysis of health outcomes and their social and health system determinants at the local level will be essential to monitor trends and advocate policies and actions that maintain the rising trend in life expectancy but avoid a grand divergence in health and longevity in England and Wales.
