# Supplementary material for: The future of life expectancy and life expectancy inequalities in England and Wales: Bayesian spatiotemporal forecasting
Source: Lancet. 2015 Jul 11;386(9989):163–70. doi: 10.1016/S0140-6736(15)60296-3 (PMC4502253; doi:10.1016/S0140-6736(15)60296-3)
Supplement: Supplementary appendix [file mmc1.pdf]

THE LANCET

Supplementary appendix

This appendix formed part of the original submission and has been peer reviewed. We post it as supplied by the authors.

Supplement to: Bennett JE, Li G, Foreman K, et al. The future of life expectancy and life expectancy inequalities in England and Wales: Bayesian spatiotemporal forecasting. *Lancet* 2015; published online April 30. http://dx.doi.org/10.1016/S0140-6736(15)60296-3.

Webappendix

Table of contents

Appendix Text 1. Forecasting model specifications 2

Appendix Text 2. Measuring the performance of forecast models. 4

Appendix Text 3. WinBUGS code for Model 3. 5

Appendix Table 1: Models used for forecasting district death rates by age group, sex, and district 7

Appendix Table 2: Mean forecast error (MFE) and median absolute forecast error (MAE) for life expectancy forecasts from different forecasting models 8

Appendix Table 3: Mean forecast error (MFE) and median absolute forecast error (MAE) for life expectancy forecasts from different forecasting models over the 11 years of model testing (2002-2012).. 10

Appendix Table 4: Mean forecast error (MFE) and median absolute forecast error (MAE) for life expectancy forecasts from different forecasting models by quintile of deprivation 14

Appendix Table 5: Mean forecast error (MFE) and median absolute forecast error (MAE) and 90% coverage of forecast models for age-specific death rates from different forecasting models 16

Appendix Figure 1: Estimated in-sample (1981-2001) life expectancy and forecasted life expectancy (2002-2012) compared with life expectancy calculated from raw data for England and Wales.. 21

Appendix Figure 2: Age group intercepts (θ_a) for Model 3 including 95% credible intervals .. 23

Appendix Figure 3: Age group slopes ($\beta 1a$) for Model 3 including 95% credible intervals 25

Appendix Figure 4: Birth cohort slopes ($\beta 3, l$) for Model 3 including 95% credible intervals . 27

References.. 29

Appendix Text 1. Forecasting model specifications

As described in Methods, we formulated four models (Models 1-4) that incorporate important and established features of death rates in relation to age and birth cohort, as well as over time and space. The model specifications are provided in Appendix Table 1 and described below.

As is common for analysing mortality, death counts (y_{atd}) in age group a $(= 1, \ldots, 19)$, year t $(= 0, \ldots, T-1$ with T being the number of observation years), and district d $(= 1, \ldots, 375)$ are modelled by a Poisson distribution, $y_{atd} \sim Poisson(n_{atd} \times m_{atd})$ where n_{atd} is the population at risk and m_{atd} is death rate, modelled on the log scale.[1]

In all four models (Models 1-4), log-transformed death rates are allowed to have different levels and trends by age group as well as by district, while borrowing strength across age groups and districts as described below. α is the common intercept for death rates across age groups and districts in 1981, the first observation year. β_0 quantifies the common trend across ages and districts. θ_a and β_{1a} measure deviation from the common level and trend, respectively, by age group. Borrowing strength and smoothness over age is done using a first-order random walk prior on θ_a and β_{1a}. The first order random walk takes a general form of $\theta_a \sim N(\theta_{a-1}, \sigma_\theta^2)$, and is implemented as a one-dimensional conditional autoregressive (CAR) model, as detailed below.

The district-specific intercept u_d and slope β_{2d} measure the deviation (common across all ages) in district d of the mortality level and trend, respectively, from the common intercept (α) and trend (β_0). We modelled u_d and β_{2d} using the Besag, York, and Mollie (BYM) model so that information is shared both locally (amongst neighbouring districts) through spatially-structured random effects with a CAR prior, and globally through spatially-unstructured Gaussian random effects.[2] The spatial structure of the CAR prior is imposed through the adjacency matrix **W** of size $n \times n$ with n being number of districts. The diagonal entries of **W** are zero, and the off-diagonal entries are specified such that $w_{ij} = 1$ if districts i and j are neighbours and $w_{ij} = 0$ otherwise. The first- and second-order random walks, used for smoothing over ages and cohorts, can be formulated as one-dimensional CAR models with adjacency matrices that specify proximity in age groups or cohorts, respectively.[3] The age-district interaction term ξ_{ad} accounts for age-specific deviations in mortality in district d from that of other districts and is modelled as $N(0, \sigma_\xi^2)$.

v_{at} and h_{dt} are first-order random walks over time that allow for nonlinearity in the age and district trends that are not accounted for by other trend components. We constrained $v_{a0} = h_{d0} = 0$ so that the intercepts can be uniquely identified. Finally, ε_{atd}, modelled as $N(0, \sigma_\varepsilon^2)$, accounts for additional variability not captured by other components in the models.

Models 2-4 differ from Model 1 by each using a different way to capture nonlinearities in trends. Model 2 includes an empirically-estimated (i.e., estimated as a part of model fitting) age-specific exponent on the trend term (t^{η_a}). A cohort component on trend, which allows trends in death rates to vary by birth cohort, is introduced in Models 3 and 4 as $\beta_{3,l}$, with the birth cohort, l, calculated as $l = (1981 + t - age_a)$ where age_a is the mid-age for age group a and 1981 is the first observation year. We did not include a cohort intercept because including components for age, time, and birth cohort on mortality level makes the estimation non-identifiable.[4, 5] The

model borrows strength and ensures smoothness of trends over birth cohorts using a second-order random walk prior on $\beta_{3,l}$.[6] This prior takes the form of $\beta_{3,l} = 2\beta_{3,l-1} - \beta_{3,l-2} + \tau_l$ with $\tau_l \sim N(0, \sigma_\tau^2)$ and, as above, is implemented using the CAR formulation. In Model 4, empirically-estimated (i.e., estimated as a part of model fitting) age-specific weights (w_a) are added to the cohort component to allow the influence of the birth cohort terms to vary by age group.

Model 5 adapts the principle of the Lee-Carter model, of decomposing the age-by-time death rate matrix using a multiplicative structure, to a Bayesian spatial framework. θ_a and u_d, respectively, measure age and district deviations from the common intercept, α. θ_a and u_d allow borrowing strength over age and district via a first-order random walk and BYM priors, respectively (we also tested models without age and space smoothing but found that they had worse performance). Like the Lee-Carter model, each district has a time trend term (γ_{dt}) and a set of age-specific multiplicative adjustments (λ_a), which are common across districts. ξ_{ad} and ε_{atd} account for age-district interactions and additional variability, as defined in Models 1-4. To help identifiability, λ_a have a moderately informative prior $N(1, 0.33)$. This prior implies that in each district, death rates in most age groups have trends that are in the same direction although they can be steeper or more gradual than the overall trend for that district. It is possible for an age group to have a trend opposite those of others, but the probability is small (about 4%). We selected this prior based on the empirical evidence on age-specific mortality trends (Figure 2 of the main paper). γ_{dt} has a first-order random walk prior to smoothly capture non-linear trends.

As in the Lee-Carter model, forecasts from Model 5 are produced through a two-stage process. In stage 1, all parameters of the model presented in Appendix Table 1 are jointly estimated. In stage 2, a linear trend model is applied to the posterior estimates of γ_{dt} in order to estimate a drift parameter for each district. The district-specific drift parameters are estimated jointly, with a common Gaussian distribution. The estimated drifts, together with the other parameters (i.e. α, θ_a, λ_a, u_d and ξ_{ad}) are used to produce district specific forecasts.

Appendix Text 2. Measuring the performance of forecast models.

As described in Methods, to assess the performance of the forecasting models, we used the first 21 years of data (1981-2001) to estimate model parameters, which were then used to forecast for 2002-2012 for which data were available but withheld from the model. We compared the forecasts from each model with the withheld data, and report forecast error (FE), which measures systematic bias of forecasts, and absolute forecast error (AE), which measures any deviation of forecasts from the actual (but withheld) data, for both life expectancy and age- specific death rates. FE and AE are calculated as below:

For life expectancy	Forecast error	$LE_{td}^{(obs)} - LE_{td}^{(pred)}$
	Absolute forecast error	$\left\| LE_{td}^{(obs)} - LE_{td}^{(pred)} \right\|$
For age-specific death rates	Forecast error	$m_{atd}^{(obs)} - m_{atd}^{(pred)}$
	Absolute forecast error	$\left\| m_{atd}^{(obs)} - m_{atd}^{(pred)} \right\|$

where superscripts (*obs*) and (*pred*) denote the observed and the posterior mean of predicted quantities, respectively, and t and d denote year (2002-2012) and district, respectively. Means, medians, and percentiles reported in Appendix Tables 2-5 are calculated over districts and years.

We also report the 90% coverage of forecasts which measures how well the posterior distributions of estimated death rates coincide with the observed death rates. Coverage estimates took into account the variations both due to the Bayesian model parameters and due to the stochastic nature of death counts in each district; the latter was dealt with by adding in Poisson variation.[7, 8]

Model performance results are provided in Appendix Tables 2-5 and Appendix Figure 1.

Appendix Text 3. WinBUGS code for Model 3.

```
model {
 for (i in 1:N) {
   y[i] ~ dpois(mu[i])
   log(mu[i]) <- log(n[i]) + epsilon[age[i],LAD[i],yr[i]]
 }

 #########################################################################
 ####      priors on the overall intercept (alpha) and overall slope (beta)
 #########################################################################
 alpha ~ dnorm(0, 0.00001)
 beta ~ dnorm(0, 0.00001)

 #########################################################################
 ####      age and district specific intercepts and age-district interactions
 #########################################################################

 ####   age intercepts with RW1 prior
 theta[1:Ages] ~ car.normal(adj.age[],weights.age[],num.age[],tau_theta)
 sigma_theta ~ dunif(0,2)
 tau_theta <- pow(sigma_theta,-2)

 ####   district intercepts with BYM prior
 u[1:LADs] ~ car.normal(adj.LAD[],weights.LAD[],num.LAD[],tau_u)
 for (d in 1:LADs) {
   tmp[d] <- alpha + u[d]
   v[d] ~ dnorm(tmp[d],tau_v)
 }
 sigma_u ~ dunif(0,2)
 tau_u <- pow(sigma_u,-2)
 sigma_v ~ dunif(0,2)
 tau_v <- pow(sigma_v,-2)

 ####   age-district interactions
 for (a in 1:Ages) {
   for (d in 1:LADs) {
     mu.age_LAD[a,d] <- theta[a] + v[d]
     age_LAD[a,d] ~ dnorm(mu.age_LAD[a,d],tau_age_LAD)
 }}
 sigma_age_LAD ~ dunif(0,2)
 tau_age_LAD <- pow(sigma_age_LAD,-2)

 #########################################################################
 ####      age/cohort related time trends
 #########################################################################
 for (a in 1:Ages) {
   gamma.temp[a,1] <- 0
   gamma[a,1] <- 0
   beta_age_all[a] <- beta + beta_age[a]
   for (tt in 2:obs.Years) {
     gamma.temp[a,tt] <- gamma[a,tt-1] + beta_age_all[a] + beta_cohort[cohort.ind[a,tt]]
     gamma[a,tt] <- gamma.temp[a,tt] + add.noise[a,tt] * noise[a,tt]
     # Note: add.noise is a matrix with all entries being 1 apart from add.noise[19,1:5]=0 and
     # add.noise[1,(obs.Years-4):obs.Years]=0 to suppress the random walk part for identifiability of
```

```
    # the oldest and the youngest cohorts

    noise[a,tt] ~ dnorm(0,tau_gamma)
  }}
  sigma_gamma ~ dunif(0,2)
  tau_gamma <- pow(sigma_gamma,-2)


  ######################################################################
  ####     age slopes with RW1 prior
  ######################################################################
  beta_age[1:Ages] ~ car.normal(adj.age[],weights.age[],num.age[],tau_beta_age)
  sigma_beta_age ~ dunif(0,2)
  tau_beta_age <- pow(sigma_beta_age,-2)

  ######################################################################
  ####     district related trends
  ######################################################################
  for (d in 1:LADs) {
   gamma_LAD.temp[d,1] <- 0
   gamma_LAD[d,1] <- 0
   for (tt in 2:obs.Years) {
    gamma_LAD.temp[d,tt] <- gamma_LAD[d,tt-1] + beta_LAD[d]
    gamma_LAD[d,tt] ~ dnorm(gamma_LAD.temp[d,tt],tau_gamma_LAD)
  }}
  sigma_gamma_LAD ~ dunif(0,2)
  tau_gamma_LAD <- pow(sigma_gamma_LAD,-2)

  ######################################################################
  ####     district slopes with BYM prior
  ######################################################################
  beta_LAD_u[1:LADs] ~ car.normal(adj.LAD[],weights.LAD[],num.LAD[],tau_beta_LAD_u)
  for(d in 1:LADs) {
   beta_LAD.temp[d] ~ dnorm(beta_LAD_u[d],tau_beta_LAD)
   beta_LAD[d] <- beta_LAD.temp[d]
  }
  sigma_beta_LAD_u ~ dunif(0,2)
  tau_beta_LAD_u <- pow(sigma_beta_LAD_u,-2)
  sigma_beta_LAD ~ dunif(0,2)
  tau_beta_LAD <- pow(sigma_beta_LAD,-2)

  ######################################################################
  ####     putting all together
  ######################################################################
  for (a in 1:Ages) {
   for (d in 1:LADs) {
    epsilon[a,d,1] <- age_LAD[a,d]
    for (tt in 2:obs.Years) {
     mu.epsilon[a,d,tt] <- age_LAD[a,d] + gamma[a,tt] + gamma_LAD[d,tt]
     epsilon[a,d,tt] ~ dnorm(mu.epsilon[a,d,tt],tau_epsilon)
  }}}
  sigma_epsilon ~ dunif(0,2)
  tau_epsilon <- pow(sigma_epsilon,-2)
}
```

Appendix Table 1: Models used for forecasting district death rates by age group, sex, and district. Models are described in Appendix Text 1.

	Common terms		Age/cohort terms		Spatial terms		
**Model 1**	$\log(m_{atd}) = [\alpha + \beta_0\, t]$	+	$[\theta_a + \beta_{1a} t + v_{at}]$	+	$[u_d + \beta_{2d}\, t + h_{dt}]$	+	$\xi_{ad} + \varepsilon_{atd}$
**Model 2**	$\log(m_{atd}) = [\alpha \quad]$	+	$[\theta_a + (\beta_0 + \beta_{1a})\, t^{\eta_a} + v_{at}]$	+	$[u_d + \beta_{2d}\, t + h_{dt}]$	+	$\xi_{ad} + \varepsilon_{atd}$
**Model 3**	$\log(m_{atd}) = [\alpha + \beta_0\, t]$	+	$[\theta_a + (\beta_{1a} + \beta_{3,l})\, t + v_{at}]$	+	$[u_d + \beta_{2d}\, t + h_{dt}]$	+	$\xi_{ad} + \varepsilon_{atd}$
**Model 4**	$\log(m_{atd}) = [\alpha + \beta_0\, t]$	+	$[\theta_a + (\beta_{1a} + w_a \beta_{3,l})\, t + v_{at}]$	+	$[u_d + \beta_{2d}\, t + h_{dt}]$	+	$\xi_{ad} + \varepsilon_{atd}$
**Model 5**	$\log(m_{atd}) = [\alpha \quad]$	+	$[\theta_a + \lambda_a \gamma_{dt}]$	+	u_d	+	$\xi_{ad} + \varepsilon_{atd}$

Appendix Table 2: Mean forecast error (MFE) and median absolute forecast error (MAE) for life expectancy forecasts from different forecasting models. See Appendix Table 3 for errors over time. Numbers in brackets show the 5th, 25th, 75th, and 95th percentiles of forecast error (FE) and absolute forecast error (AE) across districts and forecast years.

A positive FE indicates underestimation of future life expectancy and vice versa.

	Male		Female	
	FE	AE	FE	AE
	In-sample (years with data, 1981-2001)			
Model 1	0.025 (-0.951,-0.339,0.393,1.002)	0.365 (0.035,0.169,0.645,1.233)	0.047 (-0.980,-0.347,0.412,1.161)	0.378 (0.033,0.175,0.697,1.333)
Model 2	0.057 (-0.941,-0.319,0.429,1.055)	0.381 (0.035,0.170,0.664,1.290)	0.088 (-0.896,-0.304,0.443,1.181)	0.375 (0.034,0.174,0.679,1.314)
Model 3	0.025 (-0.946,-0.338,0.388,1.001)	0.364 (0.034,0.171,0.641,1.233)	0.051 (-0.991,-0.352,0.422,1.191)	0.390 (0.035,0.176,0.711,1.363)
Model 4	0.030 (-0.965,-0.348,0.404,1.037)	0.380 (0.034,0.172,0.662,1.261)	0.054 (-0.970,-0.340,0.419,1.163)	0.378 (0.034,0.174,0.694,1.322)
Model 5	0.028 (-0.944,-0.338,0.388,1.002)	0.366 (0.032,0.163,0.645,1.227)	0.051 (-0.938,-0.333,0.402,1.136)	0.364 (0.032,0.169,0.673,1.302)
	Out-sample (forecasted years, 2002-2012)			
Model 1	0.433 (-1.011,-0.213,1.038,2.052)	0.641 (0.060,0.293,1.152,2.069)	0.262 (-1.208,-0.398,0.802,2.003)	0.602 (0.056,0.286,1.081,2.048)
Model 2	0.405 (-1.053,-0.228,1.011,2.010)	0.645 (0.056,0.295,1.137,2.041)	0.267 (-1.216,-0.395,0.834,2.013)	0.608 (0.058,0.295,1.089,2.048)
Model 3	0.012 (-1.349,-0.561,0.556,1.445)	0.559 (0.060,0.265,0.959,1.703)	0.009 (-1.427,-0.616,0.540,1.715)	0.580 (0.056,0.275,1.025,1.876)
Model 4	0.419 (-1.025,-0.218,1.023,2.029)	0.638 (0.058,0.291,1.136,2.041)	0.121 (-1.306,-0.516,0.645,1.848)	0.576 (0.053,0.281,1.034,1.929)
Model 5	0.827 (-0.685, 0.146,1.459,2.563)	0.841 (0.079,0.407,1.471,2.563)	0.531 (-0.983,-0.140,1.112,2.269)	0.658 (0.062,0.303,1.226,2.274)

Appendix Table 3: Mean forecast error (MFE) and median absolute forecast error (MAE) for life expectancy forecasts from different forecasting models over the 11 years of model testing (2002-2012). Numbers in brackets show the 5th, 25th, 75th, and 95th percentiles of forecast error (FE) and absolute forecast error (AE) across districts and forecast years.

A positive FE indicates underestimation of future life expectancy and vice versa.

	Male		Female	
	FE	AE	FE	AE
	2002			
Model 1	-0.083 (-1.194,-0.572,0.342,1.124)	0.496 (0.057,0.231,0.824,1.419)	0.036 (-1.332,-0.491,0.513,1.546)	0.504 (0.040,0.215,0.922,1.718)
Model 2	-0.054 (-1.156,-0.579,0.388,1.195)	0.480 (0.054,0.223,0.832,1.497)	0.075 (-1.351,-0.444,0.573,1.588)	0.512 (0.042,0.219,0.905,1.694)
Model 3	-0.136 (-1.238,-0.625,0.292,1.067)	0.481 (0.040,0.221,0.874,1.309)	0.020 (-1.349,-0.511,0.504,1.534)	0.509 (0.034,0.218,0.929,1.721)
Model 4	-0.080 (-1.183,-0.566,0.348,1.130)	0.487 (0.058,0.228,0.818,1.430)	0.023 (-1.346,-0.512,0.503,1.542)	0.505 (0.036,0.220,0.929,1.718)
Model 5	0.210 (-0.873,-0.306,0.692,1.522)	0.507 (0.044,0.251,0.829,1.580)	0.254 (-1.193,-0.236,0.705,1.682)	0.510 (0.032,0.189,0.940,1.745)
	2003			
Model 1	-0.157 (-1.345,-0.697,0.335,1.108)	0.518 (0.054,0.248,0.885,1.464)	-0.308 (-1.448,-0.837,0.154,1.063)	0.574 (0.055,0.267,0.989,1.551)
Model 2	-0.140 (-1.311,-0.668,0.369,1.188)	0.528 (0.059,0.247,0.927,1.577)	-0.272 (-1.448,-0.772,0.186,1.082)	0.559 (0.054,0.272,0.989,1.550)
Model 3	-0.265 (-1.459,-0.813,0.223,0.990)	0.543 (0.072,0.242,0.949,1.566)	-0.347 (-1.497,-0.870,0.107,1.039)	0.581 (0.074,0.291,1.001,1.590)
Model 4	-0.153 (-1.356,-0.696,0.339,1.107)	0.519 (0.046,0.244,0.896,1.464)	-0.338 (-1.478,-0.864,0.117,1.038)	0.589 (0.075,0.284,1.003,1.586)
Model 5	0.155 (-1.121,-0.395,0.663,1.397)	0.541 (0.060,0.251,0.899,1.488)	-0.080 (-1.294,-0.579,0.401,1.231)	0.506 (0.032,0.211,0.872,1.501)
	2004			
Model 1	0.067 (-1.207,-0.377,0.567,1.403)	0.479 (0.052,0.233,0.842,1.544)	0.027 (-1.29,-0.495,0.494,1.467)	0.495 (0.029,0.220,0.856,1.657)
Model 2	0.070 (-1.225,-0.440,0.520,1.367)	0.494 (0.037,0.203,0.871,1.524)	0.0460 (-1.26,-0.480,0.54,1.529)	0.493 (0.051,0.231,0.913,1.693)
Model 3	-0.102 (-1.365,-0.534,0.396,1.216)	0.490 (0.055,0.254,0.860,1.582)	-0.041 (-1.368,-0.558,0.423,1.418)	0.483 (0.045,0.207,0.896,1.655)
Model 4	0.071 (-1.187,-0.361,0.569,1.384)	0.471 (0.049,0.236,0.852,1.537)	-0.022 (-1.359,-0.541,0.446,1.438)	0.486 (0.041,0.199,0.888,1.670)
Model 5	0.399 (-0.811,-0.113,0.899,1.783)	0.573 (0.072,0.258,0.976,1.804)	0.265 (-1.057,-0.214,0.736,1.627)	0.527 (0.056,0.236,0.904,1.704)
	2005			
Model 1	0.250 (-1.016,-0.277,0.713,1.532)	0.534 (0.053,0.254,0.931,1.641)	-0.009 (-1.382,-0.538,0.548,1.37)	0.542 (0.062,0.285,0.915,1.672)
Model 2	0.247 (-0.992,-0.284,0.763,1.626)	0.538 (0.055,0.254,0.935,1.728)	0.007 (-1.365,-0.509,0.546,1.496)	0.533 (0.054,0.274,0.886,1.737)
Model 3	0.014 (-1.252,-0.494,0.465,1.273)	0.486 (0.034,0.213,0.871,1.531)	-0.114 (-1.47,-0.644,0.436,1.256)	0.552 (0.070,0.301,0.908,1.749)
Model 4	0.253 (-1.017,-0.267,0.712,1.513)	0.523 (0.052,0.246,0.927,1.647)	-0.08 (-1.428,-0.613,0.489,1.272)	0.554 (0.051,0.271,0.915,1.722)
Model 5	0.602 (-0.685,0.072,1.100,1.951)	0.678 (0.071,0.352,1.139,1.951)	0.239 (-1.309,-0.244,0.742,1.553)	0.504 (0.077,0.261,0.943,1.701)

	Male		Female	
	FE	AE	FE	AE
	2006			
Model 1	0.339 (-0.983,-0.235,0.921,1.558)	0.593 (0.056,0.264,1.034,1.662)	0.181 (-1.297,-0.410,0.639,1.722)	0.569 (0.045,0.256,0.982,1.899)
Model 2	0.328 (-1.112,-0.244,0.909,1.652)	0.622 (0.056,0.269,1.057,1.680)	0.189 (-1.236,-0.409,0.707,1.701)	0.592 (0.055,0.291,0.980,1.918)
Model 3	0.029 (-1.289,-0.543,0.605,1.244)	0.564 (0.056,0.255,0.896,1.645)	0.032 (-1.451,-0.560,0.503,1.559)	0.523 (0.082,0.264,0.987,1.835)
Model 4	0.338 (-0.983,-0.232,0.909,1.550)	0.593 (0.063,0.264,1.027,1.672)	0.085 (-1.407,-0.491,0.557,1.623)	0.543 (0.059,0.277,0.982,1.854)
Model 5	0.710 (-0.651,0.170,1.246,2.011)	0.783 (0.062,0.406,1.271,2.034)	0.439 (-0.973,-0.134,0.953,1.940)	0.603 (0.075,0.287,1.079,1.944)
	2007			
Model 1	0.344 (-1.053,-0.207,0.909,1.835)	0.603 (0.034,0.223,1.058,1.848)	0.183 (-1.117,-0.463,0.728,1.922)	0.625 (0.058,0.318,0.988,1.979)
Model 2	0.327 (-1.055,-0.225,0.908,1.890)	0.619 (0.051,0.248,1.047,1.908)	0.196 (-1.110,-0.468,0.783,1.950)	0.599 (0.089,0.323,1.000,1.991)
Model 3	-0.045 (-1.448,-0.590,0.524,1.421)	0.555 (0.058,0.282,1.007,1.696)	-0.020 (-1.34,-0.658,0.534,1.713)	0.609 (0.074,0.327,1.005,1.840)
Model 4	0.338 (-1.069,-0.219,0.889,1.808)	0.601 (0.037,0.238,1.057,1.831)	0.059 (-1.254,-0.576,0.613,1.804)	0.586 (0.087,0.333,1.005,1.857)
Model 5	0.737 (-0.740,0.141,1.306,2.245)	0.765 (0.081,0.370,1.308,2.245)	0.451 (-0.796,-0.200,1.030,2.038)	0.593 (0.054,0.286,1.118,2.060)
	2008			
Model 1	0.447 (-0.799,-0.144,0.992,1.955)	0.572 (0.054,0.276,1.081,1.955)	0.150 (-1.241,-0.472,0.688,1.774)	0.581 (0.068,0.274,1.049,1.911)
Model 2	0.410 (-0.896,-0.194,0.965,1.917)	0.590 (0.050,0.296,1.069,1.917)	0.147 (-1.257,-0.506,0.705,1.794)	0.582 (0.062,0.275,1.123,1.818)
Model 3	-0.028 (-1.286,-0.636,0.53,1.478)	0.583 (0.062,0.266,0.932,1.718)	-0.118 (-1.537,-0.752,0.421,1.537)	0.606 (0.051,0.284,1.082,1.890)
Model 4	0.435 (-0.822,-0.173,0.997,1.942)	0.582 (0.056,0.277,1.077,1.942)	-0.006 (-1.422,-0.63,0.541,1.644)	0.587 (0.038,0.260,1.075,1.862)
Model 5	0.860 (-0.454,0.218,1.396,2.362)	0.819 (0.069,0.393,1.406,2.362)	0.429 (-0.965,-0.217,1.016,2.018)	0.645 (0.072,0.317,1.120,2.077)
	2009			
Model 1	0.650 (-0.815,0.037,1.276,2.190)	0.750 (0.071,0.351,1.334,2.194)	0.561 (-0.963,-0.137,1.168,2.166)	0.669 (0.072,0.359,1.215,2.166)
Model 2	0.598 (-0.939,-0.044,1.241,2.219)	0.764 (0.064,0.333,1.288,2.243)	0.556 (-1.029,-0.133,1.157,2.266)	0.701 (0.059,0.329,1.265,2.266)
Model 3	0.083 (-1.410,-0.540,0.712,1.573)	0.625 (0.059,0.299,1.026,1.730)	0.218 (-1.338,-0.470,0.814,1.823)	0.643 (0.065,0.296,1.057,1.996)
Model 4	0.629 (-0.861,0.006,1.264,2.138)	0.739 (0.080,0.335,1.311,2.148)	0.370 (-1.183,-0.316,0.984,1.971)	0.641 (0.087,0.316,1.133,1.982)
Model 5	1.085 (-0.480,0.439,1.678,2.689)	1.075 (0.115,0.535,1.680,2.689)	0.851 (-0.839,0.084,1.484,2.385)	0.981 (0.074,0.466,1.506,2.385)

	Male		Female	
	FE	AE	FE	AE
	2010			
Model 1	0.853 (-0.666,0.307,1.373,2.238)	0.941 (0.096,0.495,1.389,2.238)	0.556 (-0.899,-0.096,1.104,2.293)	0.676 (0.054,0.289,1.172,2.293)
Model 2	0.792 (-0.759,0.208,1.322,2.225)	0.882 (0.064,0.471,1.333,2.225)	0.550 (-0.976,-0.069,1.117,2.189)	0.656 (0.054,0.303,1.178,2.189)
Model 3	0.186 (-1.333,-0.375,0.689,1.646)	0.581 (0.082,0.296,0.987,1.752)	0.127 (-1.324,-0.524,0.675,1.859)	0.592 (0.071,0.294,0.982,1.931)
Model 4	0.823 (-0.699,0.275,1.338,2.270)	0.955 (0.087,0.457,1.354,2.270)	0.326 (-1.110,-0.321,0.881,2.048)	0.582 (0.064,0.310,1.033,2.048)
Model 5	1.309 (-0.207,0.721,1.822,2.809)	1.336 (0.208,0.741,1.822,2.809)	0.857 (-0.616,0.210,1.455,2.680)	0.846 (0.106,0.434,1.476,2.680)
	2011			
Model 1	1.088 (-0.468,0.466,1.673,2.639)	1.118 (0.145,0.558,1.673,2.639)	0.885 (-0.644,0.14,1.568,2.832)	0.822 (0.105,0.410,1.569,2.832)
Model 2	1.016 (-0.571,0.342,1.587,2.562)	1.040 (0.129,0.513,1.596,2.562)	0.868 (-0.786,0.103,1.493,2.875)	0.842 (0.093,0.452,1.503,2.875)
Model 3	0.316 (-1.199,-0.323,0.879,1.844)	0.646 (0.071,0.326,1.108,1.975)	0.359 (-1.165,-0.384,1.029,2.305)	0.712 (0.033,0.290,1.198,2.332)
Model 4	1.047 (-0.482,0.414,1.623,2.589)	1.073 (0.131,0.522,1.623,2.589)	0.613 (-0.917,-0.132,1.287,2.548)	0.739 (0.076,0.337,1.340,2.548)
Model 5	1.566 (-0.034,0.881,2.170,3.113)	1.527 (0.198,0.888,2.170,3.113)	1.197 (-0.476,0.457,1.908,3.052)	1.116 (0.116,0.544,1.908,3.052)
	2012			
Model 1	0.965 (-0.450,0.336,1.598,2.553)	0.956 (0.125,0.436,1.615,2.553)	0.619 (-0.841,-0.069,1.216,2.462)	0.755 (0.088,0.329,1.267,2.462)
Model 2	0.857 (-0.651,0.198,1.518,2.475)	0.886 (0.079,0.431,1.541,2.475)	0.580 (-1.032,-0.112,1.204,2.366)	0.694 (0.084,0.357,1.268,2.366)
Model 3	0.080 (-1.398,-0.571,0.682,1.714)	0.614 (0.069,0.319,1.042,1.844)	-0.013 (-1.490,-0.696,0.586,1.819)	0.639 (0.056,0.296,1.143,1.929)
Model 4	0.911 (-0.538,0.291,1.513,2.515)	0.928 (0.089,0.431,1.545,2.515)	0.302 (-1.178,-0.387,0.910,2.154)	0.614 (0.038,0.315,1.114,2.164)
Model 5	1.465 (-0.200,0.777,2.139,3.348)	1.412 (0.219,0.794,2.139,3.348)	0.942 (-0.529,0.206,1.585,2.855)	0.952 (0.089,0.379,1.606,2.855)

Appendix Table 4: Mean forecast error (MFE) and median absolute forecast error (MAE) for life expectancy forecasts from different forecasting models by quintile of deprivation. Numbers in brackets show the 5th, 25th, 75th, and 95th percentiles of forecast error (FE) and absolute forecast error (AE) across districts and forecast years.

A positive FE indicates underestimation of future life expectancy and vice versa.

	Male		Female	
	FE	AE	FE	AE
	Q1 (least deprived)			
Model 1	0.746 (-0.785,0.143,1.369,2.266)	0.849 (0.078,0.412,1.389,2.266)	0.662 (-0.935,-0.054,1.262,2.550)	0.731 (0.067,0.345,1.326,2.550)
Model 2	0.741 (-0.764,0.120,1.356,2.223)	0.811 (0.074,0.411,1.370,2.223)	0.686 (-0.939,-0.071,1.276,2.537)	0.740 (0.073,0.348,1.342,2.537)
Model 3	0.292 (-1.126,-0.258,0.839,1.644)	0.592 (0.057,0.291,1.027,1.703)	0.413 (-1.112,-0.268,0.985,2.171)	0.649 (0.063,0.300,1.153,2.187)
Model 4	0.702 (-0.810,0.114,1.327,2.186)	0.822 (0.085,0.377,1.357,2.186)	0.523 (-1.028,-0.169,1.100,2.289)	0.662 (0.059,0.302,1.205,2.289)
Model 5	1.024 (-0.516,0.379,1.636,2.584)	1.063 (0.127,0.522,1.639,2.584)	0.602 (-1.009,-0.098,1.187,2.488)	0.701 (0.071,0.325,1.257,2.488)
	Q2			
Model 1	0.438 (-0.989,-0.213,1.059,2.049)	0.676 (0.063,0.307,1.192,2.053)	0.383 (-1.164,-0.278,0.982,2.148)	0.617 (0.041,0.295,1.182,2.163)
Model 2	0.414 (-1.038,-0.173,1.001,1.953)	0.635 (0.078,0.286,1.134,1.960)	0.429 (-1.086,-0.212,1.009,2.093)	0.625 (0.064,0.290,1.166,2.098)
Model 3	-0.003 (-1.421,-0.59,0.587,1.447)	0.590 (0.059,0.263,1.006,1.722)	0.132 (-1.349,-0.486,0.683,1.828)	0.601 (0.051,0.279,1.047,1.906)
Model 4	0.405 (-1.050,-0.240,1.037,2.003)	0.660 (0.062,0.296,1.173,2.029)	0.242 (-1.277,-0.397,0.826,1.916)	0.593 (0.045,0.286,1.085,2.000)
Model 5	0.761 (-0.715,0.110,1.387,2.389)	0.788 (0.089,0.408,1.406,2.389)	0.454 (-1.197,-0.219,1.062,2.253)	0.639 (0.059,0.303,1.275,2.271)
	Q3			
Model 1	0.350 (-1.128,-0.246,0.946,1.897)	0.617 (0.059,0.281,1.087,1.936)	0.181 (-1.270,-0.410,0.707,1.761)	0.581 (0.050,0.256,1.014,1.862)
Model 2	0.327 (-1.110,-0.272,0.914,1.872)	0.613 (0.052,0.292,1.105,1.899)	0.172 (-1.256,-0.409,0.732,1.679)	0.563 (0.040,0.261,1.005,1.789)
Model 3	-0.079 (-1.399,-0.618,0.463,1.306)	0.553 (0.049,0.234,0.916,1.642)	-0.075 (-1.466,-0.643,0.451,1.408)	0.549 (0.058,0.280,0.985,1.799)
Model 4	0.333 (-1.138,-0.247,0.912,1.870)	0.608 (0.056,0.282,1.101,1.914)	0.039 (-1.356,-0.540,0.544,1.556)	0.541 (0.067,0.264,0.962,1.821)
Model 5	0.718 (-0.803,0.110,1.309,2.298)	0.801 (0.089,0.425,1.355,2.313)	0.391 (-1.053,-0.242,0.991,2.004)	0.619 (0.061,0.283,1.127,2.044)
	Q4			
Model 1	0.246 (-1.057,-0.350,0.806,1.817)	0.550 (0.044,0.269,1.031,1.841)	0.043 (-1.291,-0.527,0.580,1.523)	0.558 (0.057,0.264,0.944,1.648)
Model 2	0.204 (-1.141,-0.444,0.786,1.828)	0.589 (0.050,0.265,1.024,1.879)	0.045 (-1.283,-0.546,0.556,1.548)	0.550 (0.051,0.280,0.938,1.742)
Model 3	-0.160 (-1.431,-0.668,0.369,1.150)	0.546 (0.062,0.270,0.896,1.633)	-0.211 (-1.526,-0.763,0.280,1.177)	0.544 (0.051,0.250,0.953,1.645)
Model 4	0.247 (-1.077,-0.345,0.803,1.784)	0.549 (0.050,0.269,1.029,1.854)	-0.100 (-1.410,-0.659,0.410,1.321)	0.537 (0.053,0.274,0.921,1.645)
Model 5	0.669 (-0.737,-0.031,1.277,2.441)	0.704 (0.057,0.322,1.304,2.441)	0.480 (-0.919,-0.160,1.030,2.110)	0.643 (0.058,0.274,1.137,2.124)
	Q5 (most deprived)			
Model 1	0.380 (-0.931,-0.277,0.926,2.085)	0.573 (0.061,0.267,1.057,2.105)	0.036 (-1.296,-0.587,0.563,1.673)	0.578 (0.062,0.276,1.008,1.930)
Model 2	0.335 (-1.057,-0.329,0.906,2.096)	0.601 (0.048,0.265,1.078,2.096)	0.001 (-1.394,-0.615,0.578,1.719)	0.597 (0.064,0.284,1.000,1.957)
Model 3	0.007 (-1.237,-0.549,0.461,1.492)	0.519 (0.071,0.267,0.900,1.779)	-0.215 (-1.508,-0.799,0.263,1.301)	0.589 (0.065,0.272,1.017,1.889)
Model 4	0.405 (-0.922,-0.258,0.960,2.061)	0.588 (0.059,0.270,1.095,2.092)	-0.103 (-1.416,-0.714,0.392,1.493)	0.566 (0.054,0.296,1.010,1.863)
Model 5	0.959 (-0.570,0.170,1.606,3.015)	0.856 (0.079,0.401,1.606,3.015)	0.727 (-0.738,0.009,1.290,2.567)	0.698 (0.071,0.346,1.357,2.567)

Appendix Table 5: Mean forecast error (MFE) and median absolute forecast error (MAE) and 90% coverage of forecast models for age-specific death rates from different forecasting models. Numbers in brackets show the 5th, 25th, 75th, and 95th percentiles of forecast error (FE) and absolute forecast error (AE) across districts and forecast years. FE and AE are shown for death rates per 1,000.

A positive FE indicates underestimation of future death rate and vice versa.

	Male			Female		
	FE	AE	Cov90	FE	AE	Cov90
	0 year					
Model 1	0.631 (-3.690,-1.367,2.308,5.840)	1.806 (0.168,0.838,3.197,5.840)	0.914	0.306 (-3.490,-1.509,1.784,5.050)	1.639 (0.144,0.749,2.981,5.083)	0.933
Model 2	0.610 (-3.819,-1.407,2.373,5.854)	1.871 (0.168,0.847,3.298,5.870)	0.895	0.227 (-3.692,-1.625,1.763,5.050)	1.700 (0.155,0.793,3.068,5.105)	0.920
Model 3	0.230 (-4.056,-1.777,1.887,5.447)	1.827 (0.172,0.868,3.227,5.546)	0.929	-0.172 (-3.922,-2.007,1.295,4.555)	1.731 (0.186,0.819,3.113,4.758)	0.945
Model 4	0.313 (-3.984,-1.687,1.974,5.532)	1.823 (0.171,0.861,3.208,5.592)	0.928	-0.198 (-3.950,-2.032,1.263,4.528)	1.738 (0.199,0.826,3.108,4.755)	0.949
Model 5	0.959 (-3.259,-1.090,2.613,6.288)	1.835 (0.160,0.848,3.224,6.288)	0.921	0.642 (-3.097,-1.293,2.166,5.430)	1.705 (0.151,0.786,2.872,5.516)	0.929
	1-4 years					
Model 1	0.000 (-0.243,-0.197,0.162,0.558)	0.192 (0.037,0.154,0.234,0.558)	0.976	0.012 (-0.203,-0.169,0.154,0.561)	0.168 (0.037,0.141,0.208,0.561)	0.972
Model 2	-0.002 (-0.246,-0.200,0.162,0.561)	0.194 (0.036,0.156,0.235,0.561)	0.977	0.012 (-0.204,-0.171,0.153,0.563)	0.170 (0.036,0.141,0.208,0.563)	0.970
Model 3	-0.017 (-0.259,-0.213,0.144,0.539)	0.204 (0.034,0.166,0.244,0.539)	0.980	-0.012 (-0.225,-0.191,0.133,0.538)	0.187 (0.041,0.164,0.219,0.538)	0.980
Model 4	-0.013 (-0.255,-0.209,0.147,0.543)	0.201 (0.034,0.164,0.241,0.543)	0.978	-0.010 (-0.223,-0.189,0.135,0.540)	0.185 (0.041,0.162,0.218,0.540)	0.979
Model 5	-0.006 (-0.259,-0.198,0.152,0.551)	0.191 (0.039,0.149,0.241,0.551)	0.977	0.012 (-0.230,-0.165,0.150,0.574)	0.163 (0.042,0.121,0.227,0.574)	0.970
	5-9 years					
Model 1	0.004 (-0.117,-0.095,0.069,0.340)	0.094 (0.039,0.079,0.120,0.340)	0.974	-0.002 (-0.102,-0.087,0.026,0.337)	0.085 (0.054,0.074,0.101,0.337)	0.977
Model 2	0.011 (-0.111,-0.089,0.076,0.348)	0.089 (0.044,0.073,0.117,0.348)	0.969	-0.001 (-0.100,-0.086,0.029,0.336)	0.084 (0.056,0.074,0.099,0.336)	0.977
Model 3	0.001 (-0.118,-0.098,0.067,0.336)	0.096 (0.037,0.083,0.121,0.336)	0.975	-0.004 (-0.102,-0.088,0.025,0.333)	0.086 (0.052,0.077,0.101,0.333)	0.979
Model 4	0.000 (-0.120,-0.099,0.065,0.335)	0.098 (0.035,0.084,0.122,0.335)	0.976	-0.004 (-0.102,-0.088,0.025,0.333)	0.086 (0.052,0.077,0.101,0.333)	0.978
Model 5	0.002 (-0.126,-0.097,0.059,0.336)	0.095 (0.035,0.077,0.127,0.336)	0.976	-0.003 (-0.113,-0.088,0.015,0.336)	0.085 (0.042,0.069,0.109,0.336)	0.981
	10-14 years					
Model 1	-0.008 (-0.159,-0.132,0.098,0.375)	0.129 (0.029,0.110,0.153,0.375)	0.978	0.000 (-0.115,-0.098,0.062,0.339)	0.096 (0.040,0.085,0.117,0.339)	0.977
Model 2	-0.004 (-0.156,-0.128,0.103,0.381)	0.126 (0.028,0.106,0.151,0.381)	0.977	0.000 (-0.114,-0.098,0.064,0.340)	0.097 (0.041,0.086,0.116,0.340)	0.977
Model 3	0.000 (-0.150,-0.124,0.106,0.382)	0.122 (0.029,0.104,0.148,0.382)	0.977	0.005 (-0.109,-0.092,0.069,0.345)	0.091 (0.044,0.081,0.112,0.345)	0.975
Model 4	-0.010 (-0.160,-0.134,0.097,0.374)	0.130 (0.029,0.112,0.154,0.374)	0.981	0.002 (-0.112,-0.096,0.065,0.341)	0.095 (0.042,0.084,0.115,0.341)	0.977
Model 5	-0.005 (-0.161,-0.129,0.101,0.374)	0.126 (0.031,0.103,0.155,0.374)	0.978	0.000 (-0.124,-0.098,0.058,0.341)	0.096 (0.033,0.079,0.126,0.341)	0.978
	15-19 years					
Model 1	-0.071 (-0.530,-0.368,0.117,0.626)	0.254 (0.025,0.133,0.444,0.641)	0.951	-0.016 (-0.250,-0.211,0.107,0.489)	0.199 (0.024,0.105,0.234,0.489)	0.977
Model 2	-0.064 (-0.512,-0.378,0.122,0.649)	0.256 (0.031,0.134,0.444,0.657)	0.948	-0.030 (-0.264,-0.227,0.095,0.472)	0.213 (0.021,0.104,0.245,0.472)	0.976
Model 3	-0.027 (-0.487,-0.322,0.163,0.663)	0.242 (0.026,0.117,0.411,0.666)	0.954	0.005 (-0.230,-0.190,0.131,0.509)	0.182 (0.026,0.111,0.219,0.509)	0.970
Model 4	-0.072 (-0.532,-0.368,0.116,0.623)	0.254 (0.026,0.133,0.445,0.642)	0.951	-0.001 (-0.236,-0.197,0.124,0.505)	0.187 (0.026,0.110,0.223,0.505)	0.973
Model 5	-0.077 (-0.536,-0.377,0.112,0.622)	0.258 (0.024,0.134,0.450,0.646)	0.949	-0.043 (-0.278,-0.239,0.083,0.457)	0.225 (0.019,0.102,0.256,0.457)	0.981

	Male FE	Male AE	Male Cov90	Female FE	Female AE	Female Cov90
	20-24 years					
Model 1	-0.106 (-0.818,-0.479,0.154,0.940)	0.375 (0.038,0.187,0.640,0.983)	0.906	-0.004 (-0.315,-0.266,0.138,0.611)	0.250 (0.025,0.115,0.297,0.611)	0.962
Model 2	-0.075 (-0.774,-0.472,0.204,1.001)	0.384 (0.038,0.193,0.655,1.008)	0.893	-0.017 (-0.329,-0.281,0.126,0.602)	0.263 (0.021,0.115,0.308,0.602)	0.957
Model 3	-0.017 (-0.716,-0.375,0.240,1.014)	0.329 (0.036,0.165,0.595,1.018)	0.933	0.020 (-0.292,-0.240,0.164,0.641)	0.227 (0.026,0.114,0.281,0.641)	0.961
Model 4	-0.083 (-0.790,-0.453,0.177,0.954)	0.362 (0.041,0.179,0.637,0.979)	0.915	0.015 (-0.297,-0.247,0.158,0.637)	0.232 (0.026,0.113,0.284,0.637)	0.962
Model 5	-0.218 (-0.961,-0.586,0.051,0.814)	0.430 (0.044,0.219,0.685,1.046)	0.885	-0.041 (-0.356,-0.310,0.103,0.559)	0.287 (0.020,0.106,0.336,0.559)	0.964
	25-29 years					
Model 1	-0.130 (-0.860,-0.514,0.153,0.894)	0.396 (0.036,0.194,0.663,0.990)	0.902	0.005 (-0.351,-0.290,0.169,0.658)	0.267 (0.015,0.101,0.330,0.658)	0.955
Model 2	-0.107 (-0.828,-0.496,0.178,0.933)	0.388 (0.036,0.192,0.653,0.985)	0.904	-0.011 (-0.365,-0.307,0.151,0.637)	0.272 (0.019,0.100,0.342,0.637)	0.957
Model 3	-0.044 (-0.767,-0.418,0.235,0.971)	0.353 (0.036,0.173,0.618,0.981)	0.918	0.018 (-0.341,-0.277,0.184,0.670)	0.258 (0.016,0.099,0.323,0.670)	0.952
Model 4	-0.095 (-0.825,-0.472,0.190,0.923)	0.377 (0.037,0.187,0.648,0.969)	0.908	0.016 (-0.342,-0.279,0.182,0.668)	0.259 (0.016,0.099,0.323,0.668)	0.953
Model 5	-0.228 (-0.990,-0.607,0.064,0.790)	0.443 (0.043,0.216,0.694,1.082)	0.890	0.000 (-0.355,-0.299,0.167,0.654)	0.275 (0.016,0.101,0.334,0.654)	0.954
	30-34 years					
Model 1	-0.124 (-0.984,-0.524,0.196,0.922)	0.397 (0.037,0.174,0.687,1.112)	0.899	0.013 (-0.462,-0.275,0.225,0.714)	0.246 (0.025,0.115,0.420,0.714)	0.948
Model 2	-0.122 (-0.947,-0.523,0.189,0.918)	0.401 (0.031,0.184,0.669,1.059)	0.916	-0.009 (-0.486,-0.290,0.197,0.691)	0.243 (0.025,0.116,0.441,0.691)	0.955
Model 3	-0.056 (-0.902,-0.458,0.265,0.981)	0.375 (0.030,0.173,0.656,1.079)	0.908	0.010 (-0.466,-0.277,0.220,0.716)	0.247 (0.024,0.115,0.421,0.716)	0.950
Model 4	-0.082 (-0.942,-0.487,0.241,0.963)	0.385 (0.036,0.178,0.666,1.087)	0.906	0.014 (-0.462,-0.272,0.225,0.720)	0.247 (0.025,0.116,0.418,0.720)	0.950
Model 5	-0.159 (-1.050,-0.558,0.171,0.887)	0.410 (0.040,0.187,0.694,1.154)	0.897	0.025 (-0.448,-0.265,0.239,0.730)	0.247 (0.025,0.113,0.412,0.730)	0.947
	35-39 years					
Model 1	-0.037 (-0.993,-0.440,0.327,1.029)	0.390 (0.036,0.177,0.678,1.199)	0.902	-0.012 (-0.629,-0.300,0.222,0.730)	0.270 (0.024,0.126,0.460,0.765)	0.945
Model 2	-0.073 (-0.983,-0.474,0.270,0.985)	0.391 (0.038,0.185,0.675,1.159)	0.912	-0.040 (-0.668,-0.332,0.202,0.702)	0.276 (0.025,0.128,0.467,0.762)	0.946
Model 3	-0.025 (-0.984,-0.426,0.335,1.035)	0.385 (0.037,0.177,0.673,1.202)	0.904	-0.034 (-0.652,-0.322,0.200,0.710)	0.274 (0.026,0.128,0.463,0.760)	0.944
Model 4	-0.009 (-0.964,-0.413,0.351,1.056)	0.384 (0.034,0.177,0.667,1.194)	0.907	-0.021 (-0.639,-0.311,0.212,0.722)	0.273 (0.026,0.126,0.462,0.764)	0.944
Model 5	-0.047 (-1.023,-0.452,0.321,1.015)	0.395 (0.039,0.179,0.679,1.216)	0.900	0.010 (-0.602,-0.278,0.246,0.756)	0.265 (0.024,0.121,0.461,0.772)	0.944
	40-44 years					
Model 1	0.039 (-1.009,-0.418,0.466,1.228)	0.437 (0.044,0.204,0.750,1.383)	0.915	-0.007 (-0.784,-0.350,0.286,0.925)	0.327 (0.027,0.145,0.556,1.027)	0.932
Model 2	-0.039 (-1.105,-0.527,0.394,1.206)	0.473 (0.041,0.225,0.782,1.396)	0.914	-0.059 (-0.845,-0.408,0.242,0.887)	0.339 (0.033,0.159,0.574,1.031)	0.936
Model 3	-0.034 (-1.085,-0.494,0.393,1.149)	0.448 (0.040,0.206,0.758,1.395)	0.919	-0.051 (-0.819,-0.395,0.245,0.887)	0.334 (0.029,0.154,0.565,1.024)	0.929
Model 4	0.041 (-1.008,-0.416,0.468,1.232)	0.438 (0.043,0.204,0.748,1.386)	0.914	-0.036 (-0.806,-0.379,0.259,0.897)	0.331 (0.028,0.152,0.562,1.024)	0.931
Model 5	0.048 (-1.005,-0.415,0.474,1.237)	0.440 (0.046,0.206,0.753,1.385)	0.908	0.003 (-0.761,-0.335,0.298,0.926)	0.321 (0.029,0.144,0.551,1.016)	0.933

	Male			Female		
	FE	AE	Cov90	FE	AE	Cov90
	45-49 years					
Model 1	-0.052 (-1.308,-0.629,0.450,1.451)	0.554 (0.045,0.252,0.946,1.651)	0.923	-0.002 (-1.011,-0.471,0.414,1.159)	0.445 (0.036,0.201,0.750,1.334)	0.935
Model 2	-0.157 (-1.497,-0.747,0.373,1.413)	0.594 (0.055,0.274,1.003,1.729)	0.913	-0.056 (-1.087,-0.530,0.358,1.144)	0.456 (0.037,0.208,0.774,1.347)	0.933
Model 3	-0.235 (-1.514,-0.811,0.283,1.300)	0.592 (0.056,0.292,1.026,1.715)	0.913	-0.070 (-1.087,-0.538,0.346,1.103)	0.452 (0.042,0.215,0.766,1.327)	0.932
Model 4	-0.100 (-1.359,-0.679,0.402,1.417)	0.556 (0.049,0.262,0.956,1.656)	0.922	-0.054 (-1.074,-0.522,0.361,1.109)	0.449 (0.039,0.213,0.765,1.333)	0.934
Model 5	0.140 (-1.118,-0.439,0.645,1.662)	0.527 (0.048,0.238,0.928,1.735)	0.922	0.068 (-0.948,-0.394,0.481,1.240)	0.431 (0.037,0.197,0.742,1.344)	0.938
	50-54 years					
Model 1	0.124 (-1.556,-0.607,0.777,2.035)	0.697 (0.061,0.327,1.212,2.245)	0.926	-0.002 (-1.365,-0.588,0.546,1.521)	0.566 (0.057,0.272,0.968,1.699)	0.931
Model 2	0.068 (-1.729,-0.714,0.798,2.131)	0.755 (0.073,0.342,1.285,2.371)	0.904	-0.046 (-1.442,-0.651,0.525,1.520)	0.591 (0.053,0.276,1.009,1.747)	0.926
Model 3	-0.210 (-1.934,-0.969,0.483,1.702)	0.750 (0.081,0.364,1.292,2.309)	0.923	-0.127 (-1.502,-0.723,0.423,1.402)	0.582 (0.055,0.282,1.012,1.725)	0.928
Model 4	-0.018 (-1.698,-0.768,0.653,1.877)	0.708 (0.066,0.328,1.226,2.224)	0.931	-0.096 (-1.464,-0.689,0.454,1.430)	0.576 (0.050,0.275,1.000,1.716)	0.931
Model 5	0.263 (-1.435,-0.473,0.942,2.172)	0.713 (0.061,0.326,1.235,2.302)	0.938	0.114 (-1.254,-0.480,0.661,1.637)	0.566 (0.056,0.267,0.980,1.742)	0.938
	55-59 years					
Model 1	0.086 (-2.175,-0.868,0.972,2.428)	0.915 (0.077,0.418,1.575,2.874)	0.928	-0.003 (-1.802,-0.745,0.701,1.893)	0.721 (0.066,0.322,1.249,2.194)	0.925
Model 2	0.150 (-2.213,-0.846,1.099,2.708)	0.981 (0.087,0.444,1.666,3.018)	0.915	0.013 (-1.828,-0.760,0.740,1.973)	0.750 (0.061,0.350,1.300,2.278)	0.919
Model 3	-0.321 (-2.584,-1.263,0.561,2.084)	0.961 (0.091,0.443,1.652,2.896)	0.927	-0.151 (-1.945,-0.882,0.556,1.745)	0.724 (0.059,0.335,1.269,2.243)	0.926
Model 4	-0.188 (-2.444,-1.129,0.697,2.190)	0.929 (0.085,0.419,1.586,2.839)	0.930	-0.108 (-1.906,-0.846,0.587,1.786)	0.722 (0.064,0.335,1.262,2.226)	0.927
Model 5	0.247 (-1.992,-0.690,1.150,2.650)	0.925 (0.084,0.418,1.596,2.906)	0.960	0.115 (-1.693,-0.632,0.842,2.025)	0.722 (0.063,0.346,1.286,2.295)	0.940
	60-64 years					
Model 1	-0.126 (-3.321,-1.411,1.089,3.276)	1.272 (0.108,0.589,2.246,3.924)	0.916	-0.223 (-2.566,-1.193,0.676,2.297)	0.973 (0.092,0.447,1.719,2.938)	0.928
Model 2	0.1630 (-3.149,-1.236,1.389,3.712)	1.309 (0.117,0.606,2.296,4.251)	0.914	-0.073 (-2.521,-1.101,0.894,2.491)	1.002 (0.092,0.453,1.764,3.004)	0.923
Model 3	-0.266 (-3.463,-1.580,0.949,3.184)	1.316 (0.128,0.629,2.258,3.996)	0.913	-0.203 (-2.542,-1.182,0.698,2.309)	0.974 (0.093,0.449,1.718,2.947)	0.928
Model 4	-0.535 (-3.712,-1.867,0.692,2.942)	1.389 (0.124,0.656,2.383,4.089)	0.911	-0.182 (-2.514,-1.154,0.717,2.330)	0.972 (0.091,0.447,1.712,2.937)	0.930
Model 5	-0.301 (-3.545,-1.602,0.940,3.054)	1.303 (0.115,0.598,2.268,3.955)	0.969	-0.279 (-2.731,-1.268,0.647,2.303)	0.994 (0.084,0.445,1.760,3.079)	0.947
	65-69 years					
Model 1	-0.738 (-5.099,-2.557,0.944,3.880)	1.878 (0.171,0.884,3.215,5.527)	0.928	-0.531 (-3.806,-1.883,0.706,3.043)	1.395 (0.128,0.646,2.417,4.178)	0.921
Model 2	-0.069 (-4.509,-1.871,1.644,4.506)	1.765 (0.172,0.826,3.124,5.456)	0.954	-0.162 (-3.658,-1.577,1.209,3.501)	1.422 (0.140,0.660,2.423,4.331)	0.930
Model 3	-0.094 (-4.441,-1.910,1.628,4.504)	1.773 (0.165,0.821,2.995,5.336)	0.940	0.010 (-3.260,-1.318,1.260,3.506)	1.285 (0.117,0.611,2.270,4.132)	0.928
Model 4	-1.207 (-5.555,-3.041,0.501,3.467)	2.076 (0.177,0.965,3.470,5.865)	0.915	-0.009 (-3.271,-1.335,1.242,3.488)	1.285 (0.120,0.616,2.275,4.120)	0.930
Model 5	-2.277 (-6.809,-4.097,-0.482,2.370)	2.611 (0.255,1.228,4.205,6.848)	0.941	-1.535 (-4.935,-2.921,-0.233,2.054)	1.863 (0.183,0.918,3.067,5.002)	0.888

	Male			Female		
	FE	AE	Cov90	FE	AE	Cov90
	70-74 years					
Model 1	-2.565 (-8.901,-5.229,-0.142,4.149)	3.404 (0.326,1.634,5.529,9.109)	0.895	-1.805 (-6.722,-3.830,0.117,3.421)	2.498 (0.220,1.143,4.153,6.976)	0.878
Model 2	-1.845 (-7.998,-4.464,0.583,4.777)	2.874 (0.300,1.392,5.002,8.313)	0.948	-1.870 (-6.849,-3.956,0.111,3.458)	2.531 (0.267,1.250,4.294,7.148)	0.891
Model 3	-0.265 (-6.402,-2.766,2.093,6.206)	2.479 (0.215,1.149,4.395,7.727)	0.947	-0.213 (-5.074,-2.141,1.583,4.856)	1.859 (0.172,0.865,3.287,6.062)	0.929
Model 4	-2.758 (-9.131,-5.442,-0.340,4.011)	3.522 (0.369,1.703,5.711,9.295)	0.889	-0.628 (-5.440,-2.554,1.184,4.431)	1.943 (0.175,0.895,3.425,6.185)	0.930
Model 5	-5.176 (-11.781,-7.917,-2.593,1.708)	5.329 (0.658,2.927,7.944,11.781)	0.859	-3.311 (-8.276,-5.400,-1.336,2.012)	3.521 (0.418,1.866,5.493,8.282)	0.790
	75-79 years					
Model 1	-5.174 (-15.369,-9.590,-1.057,5.791)	6.183 (0.622,3.093,9.912,15.529)	0.851	-2.675 (-10.441,-5.879,0.356,5.608)	3.844 (0.383,1.909,6.398,10.691)	0.872
Model 2	-5.736 (-16.427,-10.351,-1.251,5.814)	6.692 (0.617,3.291,10.574,16.683)	0.891	-3.233 (-11.155,-6.537,-0.163,5.308)	4.251 (0.412,2.16,6.932,11.297)	0.869
Model 3	-0.534 (-9.740,-4.354,2.945,9.286)	3.696 (0.333,1.724,6.586,11.550)	0.957	-0.095 (-7.185,-2.992,2.678,7.196)	2.834 (0.235,1.310,4.991,8.804)	0.938
Model 4	-4.427 (-14.397,-8.702,-0.477,6.253)	5.613 (0.550,2.829,9.132,14.572)	0.889	-1.198 (-8.518,-4.147,1.586,6.480)	3.105 (0.288,1.420,5.333,9.245)	0.928
Model 5	-7.211 (-17.955,-11.849,-2.894,4.14)	7.687 (0.802,3.985,11.981,18.024)	0.858	-3.737 (-11.387,-6.894,-0.670,4.327)	4.406 (0.457,2.247,7.166,11.463)	0.847
	80-84 years					
Model 1	-2.264 (-18.714,-9.147,4.103,15.637)	7.101 (0.646,3.24,12.112,20.734)	0.936	-0.918 (-12.113,-5.625,3.478,11.020)	4.606 (0.492,2.194,7.962,13.955)	0.942
Model 2	-1.972 (-20.190,-9.212,5.342,16.709)	7.458 (0.661,3.552,12.66,23.003)	0.945	-0.356 (-11.428,-4.886,3.881,11.280)	4.383 (0.399,2.050,7.737,13.836)	0.966
Model 3	3.841 (-11.808,-2.461,9.824,20.324)	6.679 (0.631,3.159,11.736,21.236)	0.927	1.414 (-9.441,-3.107,5.630,12.869)	4.359 (0.440,2.104,7.633,13.927)	0.943
Model 4	0.223 (-15.574,-6.325,6.393,17.507)	6.353 (0.597,3.017,11.377,20.175)	0.952	0.545 (-10.356,-3.988,4.774,12.177)	4.388 (0.407,2.066,7.569,13.807)	0.944
Model 5	-9.283 (-26.411,-16.185,-2.549,8.789)	10.501 (1.046,5.224,16.582,26.574)	0.870	-3.975 (-15.236,-8.640,0.476,7.877)	5.605 (0.488,2.683,9.415,15.683)	0.914
	85+ years					
Model 1	-3.455 (-35.837,-17.051,8.64,31.101)	13.268 (1.188,6.118,23.309,41.159)	0.931	0.826 (-22.065,-8.157,9.675,23.903)	8.971 (0.707,4.198,15.599,27.797)	0.932
Model 2	-12.108 (-62.437,-29.757,7.026,34.800)	20.058 (1.859,9.206,34.517,64.090)	0.804	-5.222 (-35.685,-16.867,7.003,24.784)	12.564 (1.176,5.847,21.587,38.098)	0.874
Model 3	4.764 (-26.943,-7.894,17.117,37.415)	12.706 (1.256,6.121,22.451,40.877)	0.931	1.889 (-20.474,-7.144,10.509,24.767)	8.759 (0.674,4.245,15.550,27.846)	0.936
Model 4	3.401 (-27.635,-9.444,15.467,36.317)	12.415 (1.224,5.957,21.874,40.241)	0.939	0.580 (-22.199,-8.483,9.328,23.734)	8.871 (0.781,4.146,15.561,27.796)	0.937
Model 5	-11.049 (-44.149,-25.137,1.835,24.604)	16.341 (1.571,8.019,27.725,46.594)	0.821	-2.609 (-27.074,-12.134,6.831,22.517)	9.887 (0.875,4.672,16.918,29.948)	0.848

Appendix Figure 1: Estimated in-sample (1981-2001) life expectancy and forecasted life expectancy (2002-2012) compared with life expectancy calculated from raw data for England and Wales. As described in Appendix Text 2, to measure the performance of forecast models, we used the first 21 years of data (1981-2001) to estimate model parameters, which were then used to forecast for 2002-2012 for which data were available but withheld from the model.

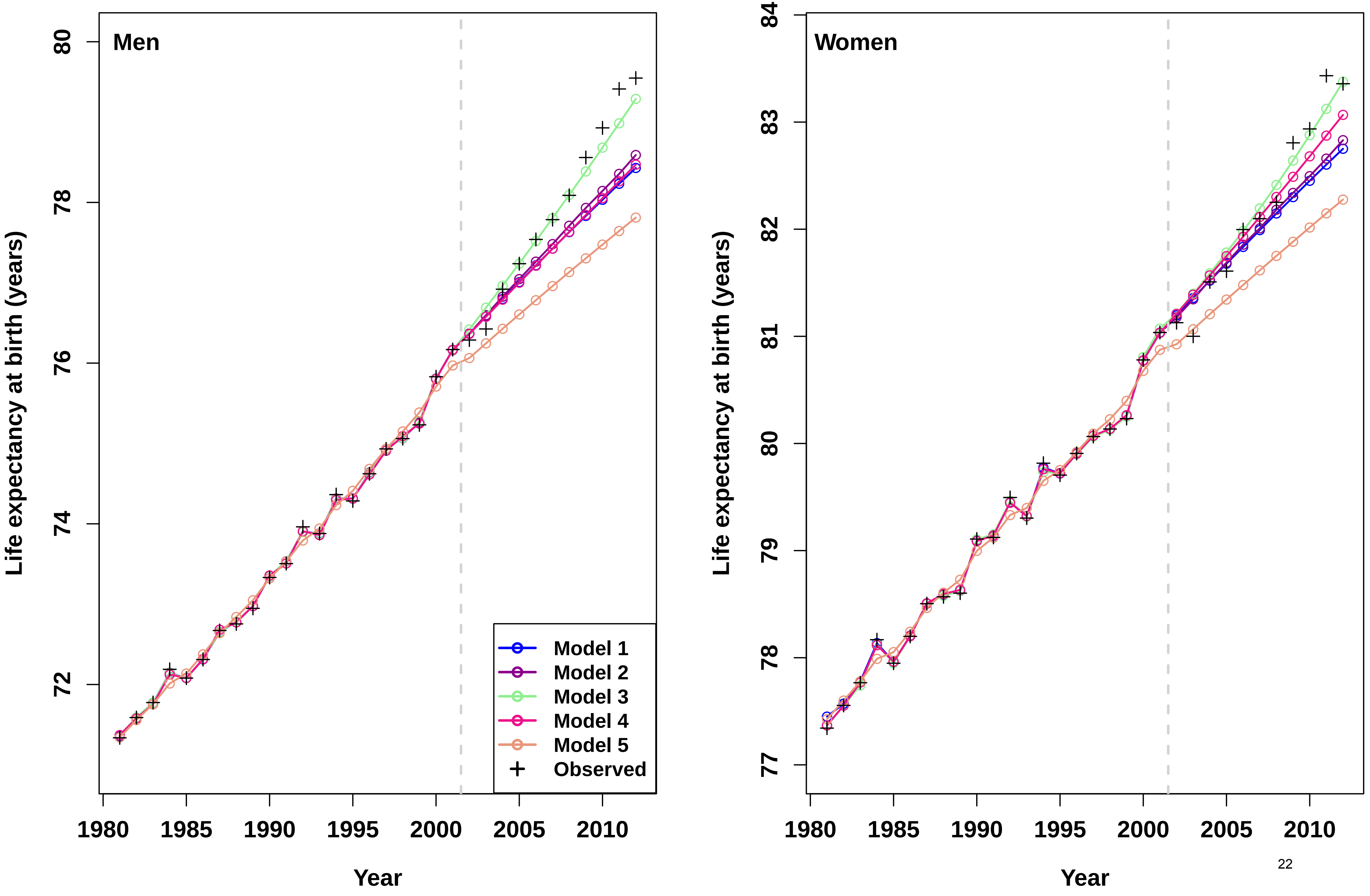

Men
Women
Life expectancy at birth (years)
Year
Model 1
Model 2
Model 3
Model 4
Model 5
Observed

Appendix Figure 2: Age group intercepts (θ_a) for Model 3 including 95% credible intervals.

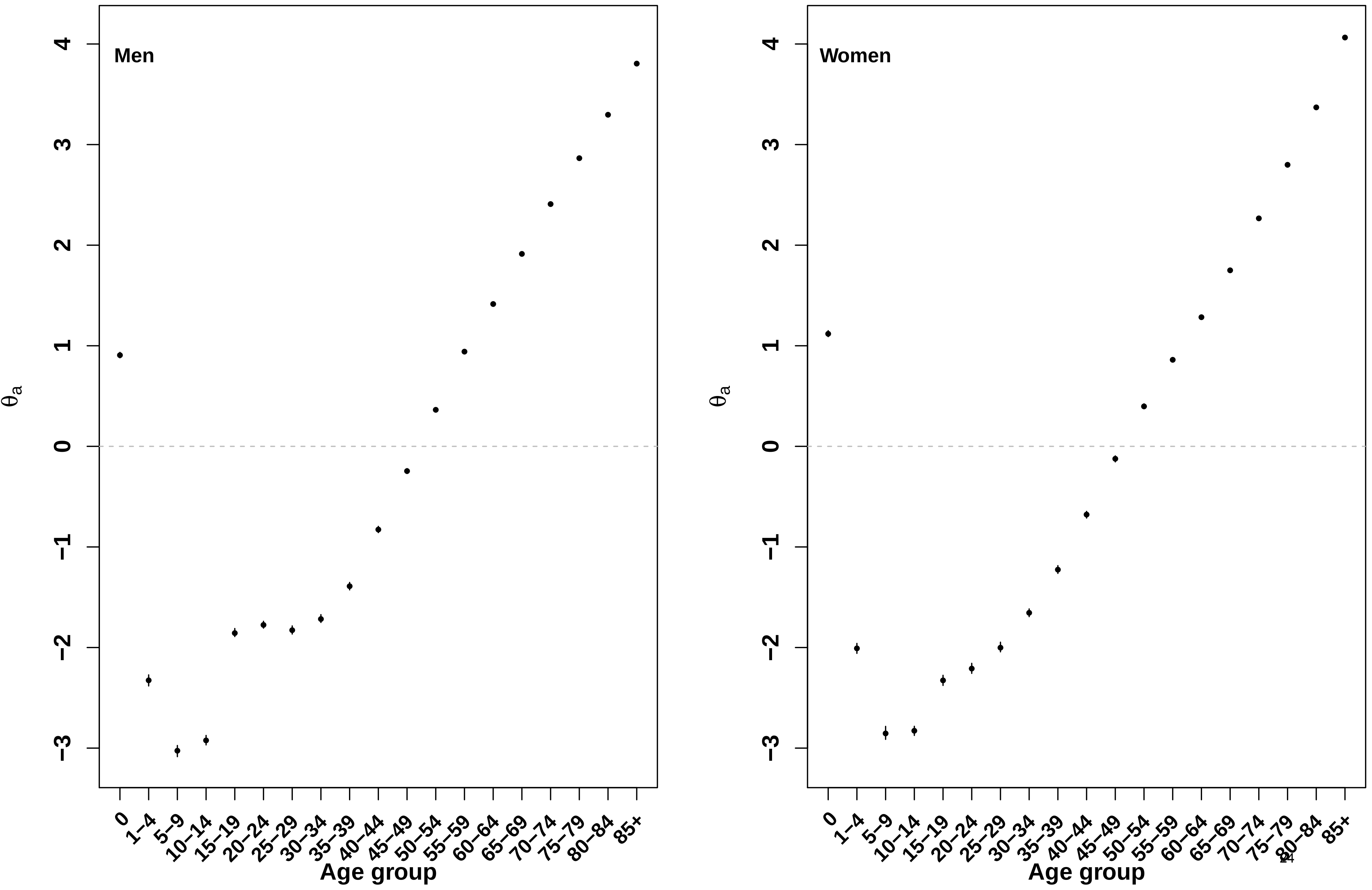
Men
Women
θ_a
Age group
0
1–4
5–9
10–14
15–19
20–24
25–29
30–34
35–39
40–44
45–49
50–54
55–59
60–64
65–69
70–74
75–79
80–84
85+
−3
−2
−1
0
1
2
3
4

Appendix Figure 3: Age group slopes (β_{1a}) for Model 3 including 95% credible intervals. Note that change in mortality is a function of age and birth cohort (Appendix Figure 4). Also note that the age group and birth cohort slopes are in addition to the common slope (β_0), which was negative for both men and women. Therefore a negative age group slope can be interpreted as, when adjusted for birth cohort, death rates for that age group declined faster than other age groups; a positive age group slope implies the opposite.

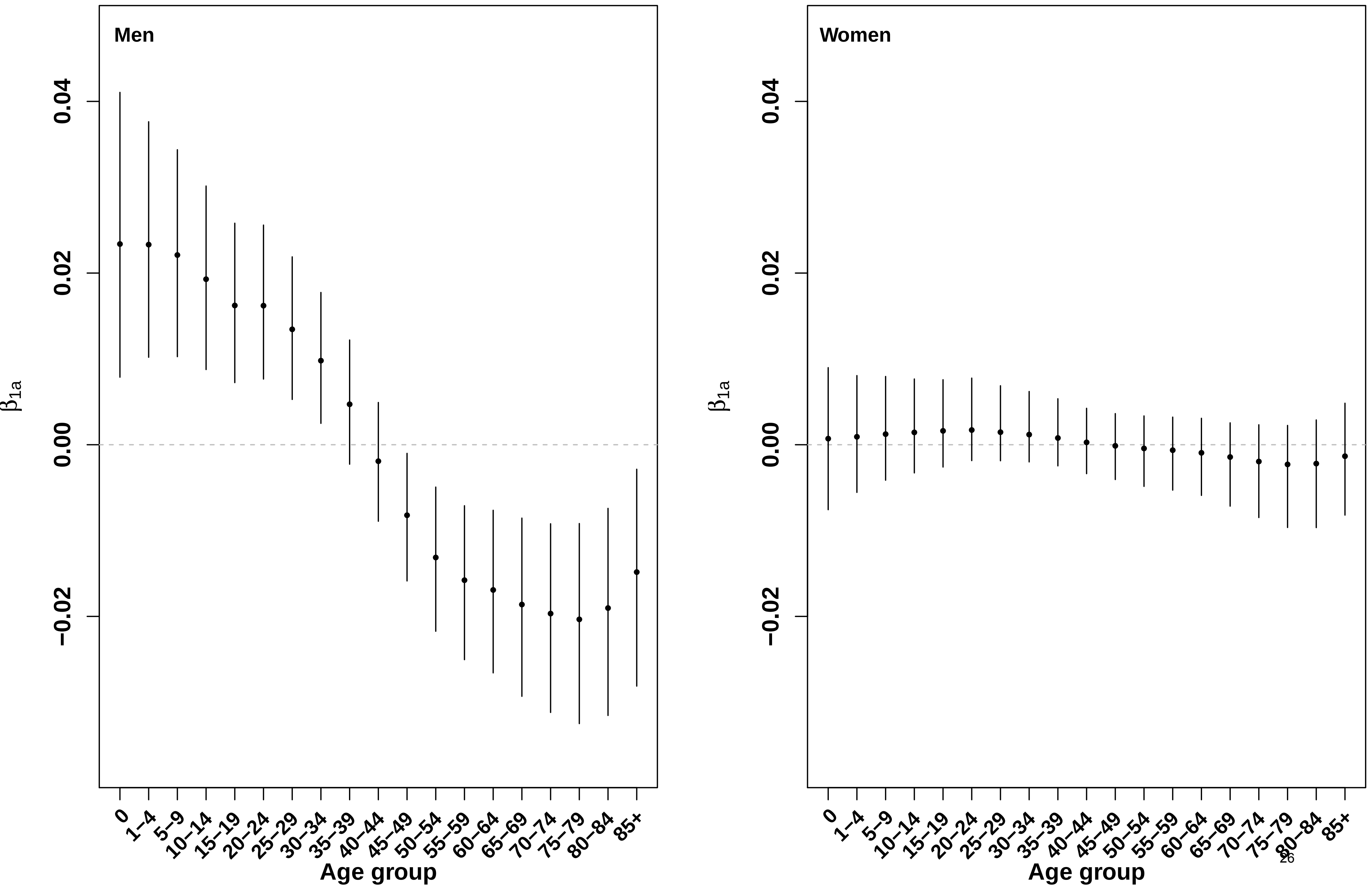
Men
Women
0.04
0.02
0.00
−0.02
β_{1a}
0
1−4
5−9
10−14
15−19
20−24
25−29
30−34
35−39
40−44
45−49
50−54
55−59
60−64
65−69
70−74
75−79
80−84
85+
Age group

Appendix Figure 4: Birth cohort slopes ($\beta_{3,l}$) for Model 3 including 95% credible intervals. Note that change in mortality is a function of age (Appendix Figure 2) and birth cohort. The cohorts born in the early 1930s, whose mortality declined faster than adjacent cohorts, are known as the "golden generation".[9]

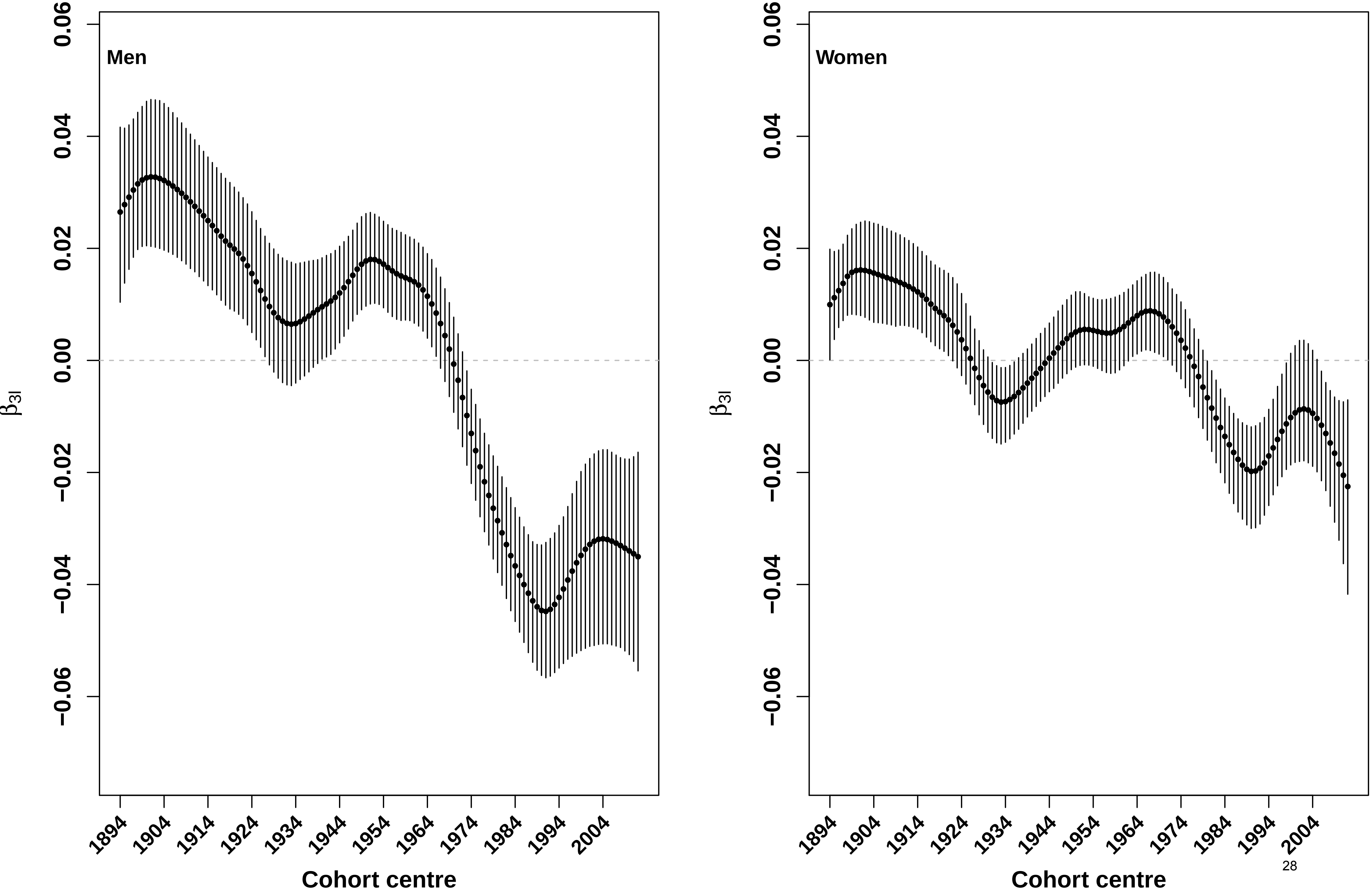
Men
Women
0.06
0.04
0.02
0.00
−0.02
−0.04
−0.06
β_{3l}
1894
1904
1914
1924
1934
1944
1954
1964
1974
1984
1994
2004
Cohort centre

References

1. McCullagh P, Nelder J. Generalized Linear Models. 2nd ed: Chapman and Hall/CRC; 1989.
2. Besag J, York J, Mollie A. Bayesian Image-Restoration, with 2 Applications in Spatial Statistics. *Ann I Stat Math* 1991; **43**(1): 1-20.
3. Fahrmeir L, Lang S. Bayesian inference for generalized additive mixed models based on Markov random field priors. *J Roy Stat Soc C-App* 2001; **50**: 201-20.
4. Clayton D, Schifflers E. Models for temporal variation in cancer rates. II: Age-period-cohort models. *Stat Med* 1987; **6**(4): 469-81.
5. Wilmoth JR, Vallin J, Caselli G. When does a cohort's mortality differ from what we might expect? *Population: English Selection* 1990; **2**: 93-126.
6. Clayton D. Generalized linear mixed models. In: Gilks WR, Richardson S, Spiegelhalter DJ, eds. Markov Chain Monte Carlo in Practice. London: Chapman & Hall; 1996: 275-301.
7. Christoffersen P. Evaluating interval forecasts. *International Economic Review* 1998; **39**(4): 841-62.
8. Brillinger DR. The natural variability of vital rates and associated statistics. *Biometrics* 1986; **42**(4): 693-734.
9. Murphy M. The ‘golden generations’ in historical context. *British Actuarial Journal Supplement* 2009; **15**: 151-84.
